# Supplementary material for: Improving the Annotation of the Venom Gland Transcriptome of Pamphobeteus verdolaga, Prospecting Novel Bioactive Peptides
Source: Toxins (Basel). 2022 Jun 15;14(6):408. doi: 10.3390/toxins14060408 (PMC9228390; doi:10.3390/toxins14060408)
Supplement: Supplementary file 1 [file toxins-14-00408-s001.zip › supplementary-Figures and Tables.pdf]

toxins

MDPI

Supplementary Materials: Improving the annotation of the venom gland transcriptome of *Pamphobeteus verdolaga*, prospecting novel bioactive peptides

Cristian Salinas-Restrepo, Elizabeth Misas, Sebastian Estrada-Gómez, Juan Carlos Quintana-Castillo, Fanny Guzman, Juan C. Calderón, Marco A. Giraldo and Cesar Segura.

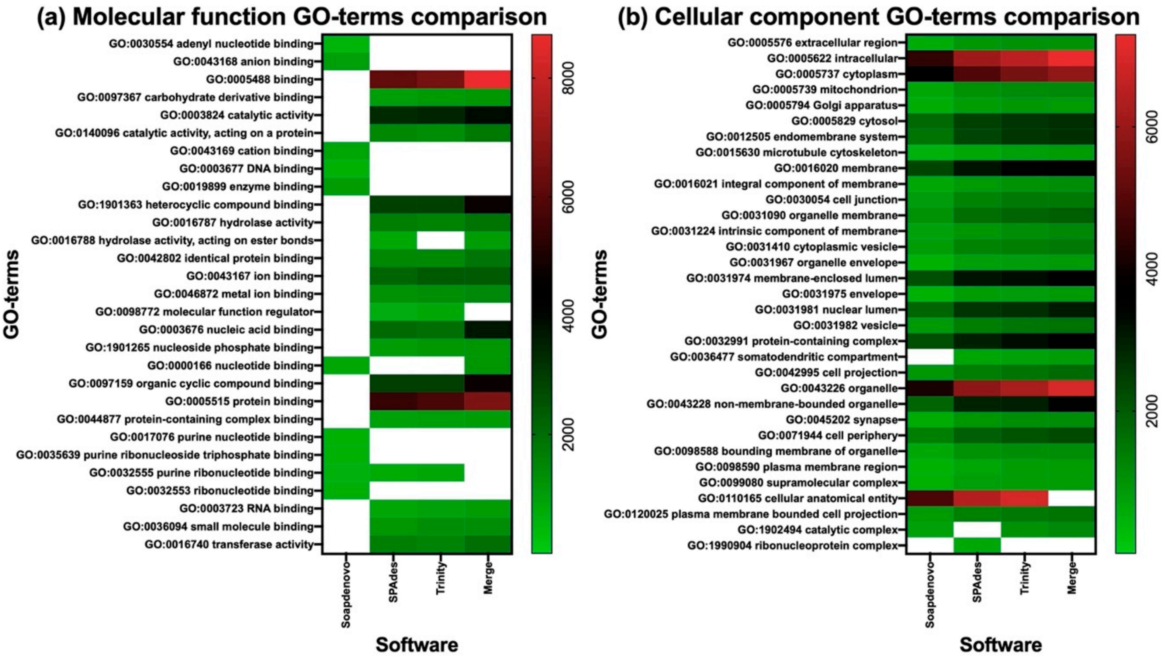

Figure S1. Comparison of the molecular function (a) and cellular component (b) non-redundant GO-terms associated to the functional annotation of the ORFs obtained from the Soapdenovo-Trans k-mer 63, SPAdes k-mer 31, Trinity k-mer 25 assemblies and the non-redundant ORF merge. Blank spaces represent absence of genes associated to the non-redundant GO-term.

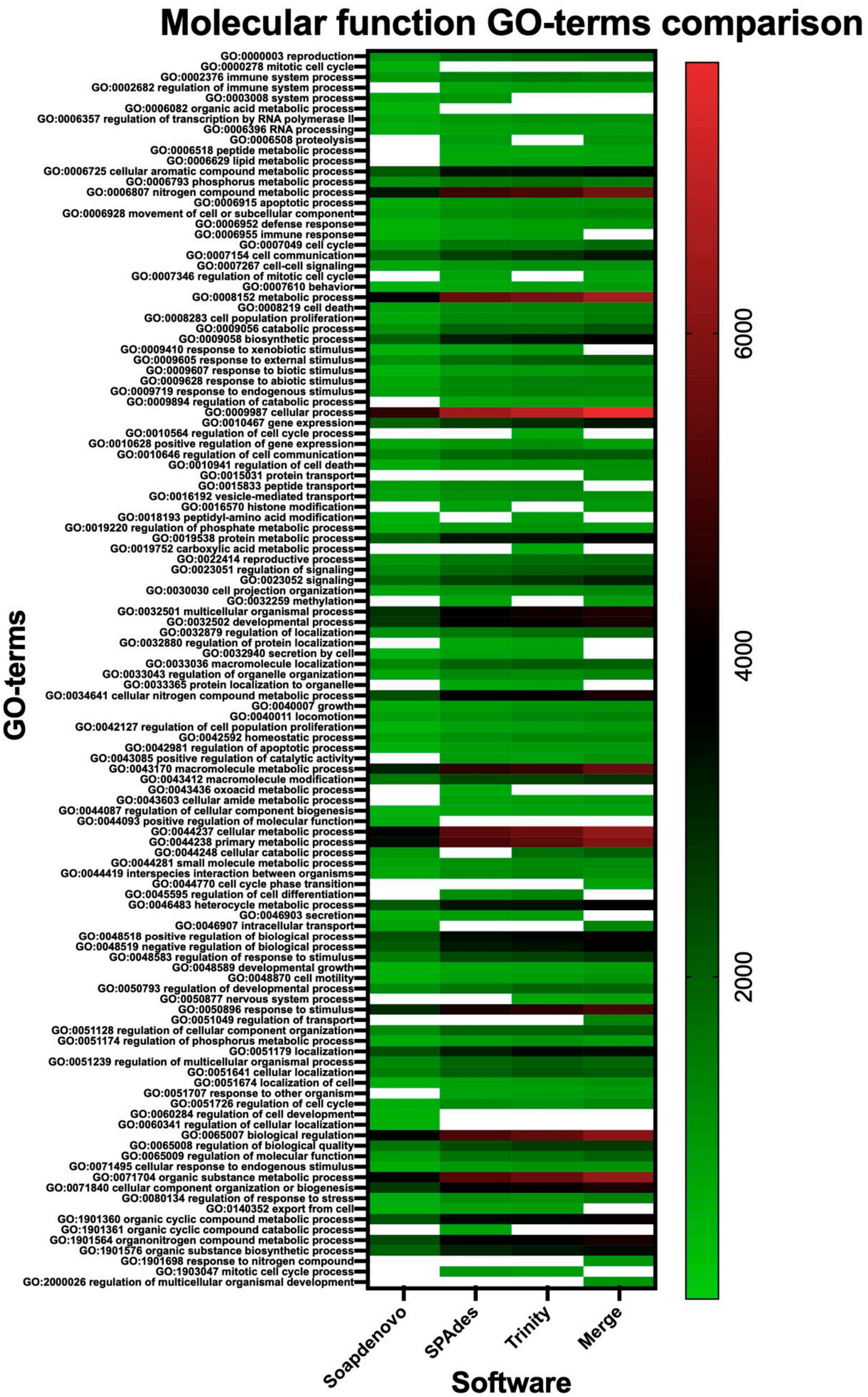

Figure S2. Comparison of the biological process non-redundant GO-terms associated to the functional annotation of the ORFs obtained from the Soapdenovo-Trans k-mer 63, SPAdes k-mer 31, Trinity k-mer 25 assemblies and the non-redundant ORF merge. Blank spaces represent absence of genes associated to the non-redundant GO-term.

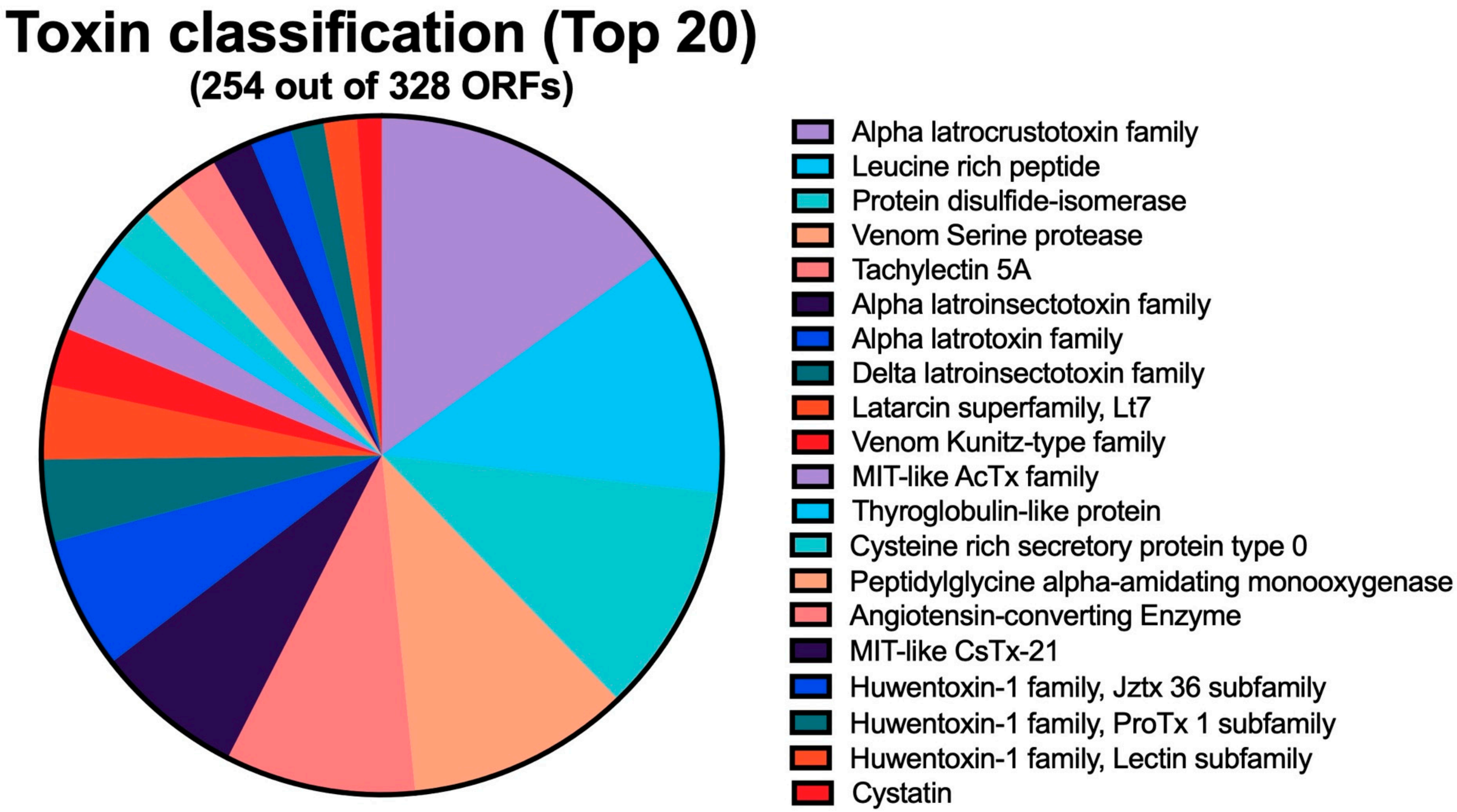

Figure S3. Top 20 families classification of the ORFs of interest from the merge annotation obtained from the BLAST (Arachnoserver + ToxProt) and HMM (hmmcompete) prediction strategies.

Table S1. List of the molecular function, cellular component and biological process GO-terms uniquely identified in the Trinity k-mer 25, SPAdes k-mer 31 and Soapdenovo-Trans k-mer 63 functional annotation.

GO Category	# of total terms	Soapdenovo-Trans	SPAdes	Trinity
**Molecular function**	29	"GO:0030554 adenyl nucleotide binding"<br>"GO:0043168 anion binding"<br>"GO:0043169 cation binding"<br>"GO:0003677 DNA binding"<br>"GO:0019899 enzyme binding"<br>"GO:0000166 nucleotide binding"<br>"GO:0017076 purine nucleotide binding"<br>"GO:0035639 purine ribonucleoside triphosphate binding"<br>"GO:0032553 ribonucleotide binding"	"GO:0016788 hydrolase activity, acting on ester bonds"	-
**Cellular component**	33	-	"GO:1990904 ribonucleoprotein complex"	-
**Biological process**	113	"GO:0000278 mitotic cell cycle"<br>"GO:0006082 organic acid metabolic process"<br>"GO:0044093 positive regulation of molecular function"<br>"GO:0046907 intracellular transport"<br>"GO:0060284 regulation of cell development"<br>"GO:0060341 regulation of cellular localization"	"GO:0006508 proteolysis"<br>"GO:0007346 regulation of mitotic cell cycle"<br>"GO:0016570 histone modification"<br>"GO:0032259 meth-ylation"<br>"GO:0043436 oxoacid metabolic process"<br>"GO:1901361 organic cyclic compound catabolic process"	"GO:0010564 regulation of cell cycle process"<br>"GO:0019752 carboxylic acid metabolic process"<br>"GO:0050877 nervous system process"

Table S2. Summary of the mature toxins from the merge transcriptome of *Pamphobeteus verdolaga* obtained with the BLAST (Arachnoserver + ToxProt) and HMM (hmmcompete) prediction strategies.

Cluster	Given name	sequence_id	HMM family_name	Blast2GO annotation	Cys	Length	Proposed mature sequence
1	U-theraphotoxin-Pv7c	Pv_sp_15215.t1	Huwentoxin-1 family, HaTx subfamily (IPR011696;PF07740;)	Kappa-theraphotoxin-Gr1a	6	35	GCGYLFGRCQNDADCCKHLGCKRIPKYCAWDGTFS
1	U-theraphotoxin-Pv7a	Pv_sp_10596.t1	Huwentoxin-1 family, Jztx 36 subfamily (IPR011696;PF07740;)	Kappa-theraphotoxin-Gr1a	6	36	DCRKMFGGCEKDENCCKHLVCKPTLNYCLWDGTFPW
1	U-theraphotoxin-Pv7b	Pv_sp_11628.t1	Huwentoxin-1 family, Jztx 36 subfamily (IPR011696;PF07740;)	Kappa-theraphotoxin-Gr1a	6	36	DCRKLFGSCEKKADCCKYLGCKRTVKYCAWDGTFPW
2	U-theraphotoxin-Pv12b	Pv_sp_12576.t1	MIT-like CsTx-22	U-scoloptoxin(18)-Er1a	10	69	ELGESCEHSSDCSSGCCMKRKSAKSQCNRKAKIGEPCSGGQIKLDLYTERCPC EKGMSICSDEIHKCLA
2	U-theraphotoxin-Pv12a	Pv_sp_11319.t1	MIT-like AcTx family (IPR020202;PF17556)	Colipase	10	73	NRAVGASCRFSSQCSSGCCVRTKPGGIRSCARKGKKGDLCSEQQIKGGAYVD YCPCEKGDGHCQTGKQSKCLA
3	U-theraphotoxin-Pv51a	Pv_sp_3139.t1	Thyroglobulin-like protein	SPARC	24	510	SGPVCGTDGRTYDSLCDIDVAKCQGHPVEFKNFGTCPERARCEAQRALLMK QGNQMFVPECNTDGSYAEVQCHKSTGYCWCVDKDGKPVRGSSVKQRRPNC SSAGRRPNNRRRTSRGRRQKKGCNSSDRAMFNSNLIDIFESEYNRLEEPPLTTS AGDDADPLTDTHQKAVVQWKFNQLDENGDNYLKKPEVRDLRRMAKKIVK PKVCAKTFAKYCDLDNDKKISRSEWSVCVGVDINNEEVNIMDQVLDDAPAV NDHRAANPSSRSLSIRPELWDSQRQHLKHDGRLNAENDGSTVELDEKEEEIQ DCETARRAALENHRQDPAAGIYIPECNKEGFYMRAQCHKSQKFCWCVHVRT GKPLKGTTTQGVKPDCESARSSTRQFKGCSFQKKQDFLDELVQSFVQEVMED AKNKSLSVEAPTHERAARWKFAYIDSNSNEVLDKREWKVFKREWQSFHKDS KQKKRLRKCWRNLPRFCDENNNQKITMDEWLMCTGITRDFKAGPAVTSG
4	U-theraphotoxin-Pv17a	Pv_sp_11450.t1	Arthropod Phospholipase A2 (IPR001211)	Group 10 secretory	10	116	SGLDFIGYGNYCGFGGEGKPVDDIDRCCKMHDICYDFAQNDDCAEDPNVVY KIKYGWQQKSFGVQCSFSQSKCMKVVCICDVRFAKCLKNYINEYNNSNKHE KDLQELLEEVQQMS

				phospholipase A2			
4	U-theraphotoxin-Pv18a	Pv_sp_12864.t1	Arthropod Phospholipase A2 (IPR001211)	Phospholipase A2 SSD387	10	122	DLLDLADMFEELTQLDPTEYIPYGNWCGYGGDGEILDRIDRCCEIHDRCYGKVSENVCSNEQVHIINYQWNRENDTITCDGNTSKCEMEACMCDRDVVLCIHKHNGDYSHEVRYVRFNQTKN
5	U-theraphotoxin-Pv52a	Pv_sp_1293.t1	Angiotensin-converting Enzyme (IPR033591)	Acetylcholinesterase	6	514	GKLVNIDGNEVTAFLGIPYAEPPVGDQRFKRSVPKQSWSGVYDTTKKPPPCMQYSTKDFPWMTKSTPSEDCLYLNVWVPGNMNFFELGFKSKPVLLWIYGGGFFSGSSELDIYNGATLAAVGKVIVVTVNYRLGSFGFLTSGTPEAPGNMGMHDQYLAMDWVKNNIKYFKGDPDMITLFGESVGGVSAGLHLISPLSRNLFRRAIMESGAPYHPLAAGNQSVMVYVSNLFATAAGCANDTVTIFTDSKKVIDCLRKTDSRQLATVESSLLEKVPYFFLPIYGDDLFPVDAITAMDKGDFKHTEVLIGNNLDEGAVLLHYISPGIFGSQHASQLDKQQAAAVITGLFQNSTNPPPQVVSEFYLNDISDTDSSTIIKAVVDSYGDFIINCPSVLFAEKFSKGGEAFYYQFTHRSSNSPWGKWKKACDFWRNYFVPSMGATHFEEVPFVFGDPVRNSSQYTAEEVTFSRTVMKIWTTFARTGVPTVPKTEGWPKFTESRRLYLELNPKRLRIGEGPH
5	U-theraphotoxin-Pv56a	Pv_tr_11548.t1	Peptidylglycine alpha-amidating monooxygenase (IPR000720)	Acetylcholinesterase	8	528	HWEGEYNATFLPPPCYQNYTIKFYWTPDVERMTEDCLYLNIWVPGSVQETKPKAVFVWIYGGAFNVGSSELDLYNGAILASRGNMVVISFNYRVGMFGFLTTSIPEAKGNMGMFDQILALKWIKANVHFFGGNSELITLAGESAGAMSAALFLLSPVTRGMVKRVILQSGGIIHPMSTHLKATMFKVAEQVSLFTGCSNNNVSLTTNPKIVIDCLKNLPPNAISEAEGLLMKIFPITASPYSGEDFMPRNPTDMLIDGDFQDTEVLFGCNRDEGSNFLVFVAPEYFGIYGAGAPTMRKRLSAYIIKTIFERTEQKNNTRDIVRFYVEGRGNRKLRNEGSDDGDETDYFKTAIYDSIGDYMILCPTVFNADFMSVREKPVYFYMFTHRPSTTLNAKWMGATHFDEVPFVFGRPLSDDKNYTEEDKTISQRMMDRWIAFTKTGNPNIEGEIEWPHYKWNEPLYLDINAEEKIKLKPDDYRCEFWRARFGSTLDHFRSDTNVDTSIGTVSTKKVPFVLLMMCVMLLTMTRT
6	U-theraphotoxin-Pv20a	Pv_tr_1740.t1	Venom Kunitz-type family, Long Kunitz domain containing protein (IPR002223;IPR020901;PF00014;)	Tissue factor pathway inhibitor	10	136	YCPGDLDLGDICSLGPEPGVPGTNISCLAYFPRYYFNGLSGECDIFIFGGCGGNHNNFYTKEECEKFCPPVPQIPFDPTECGQYPEPGDCRAYLQRFHYDKESGDCKEFTWRMWWKQKQLQDAGRMQRHMWWSGDV
6	U-theraphotoxin-Pv55a	Pv_tr_3827.t1	Venom Kunitz-type family, Native Kunitz toxin subfamily (IPR002223;IPR020901;PF00014;)	Tissue factor pathway inhibitor	34	527	GPVCSDPKISPRVCKDECVKVTCPQHPEAICLADPCHNCVISFVSVDGTPVTCEDKCRQPLTAGNCRSFLPHYHFNQSSNRCEQFIYGGCHGNDNNFETLETCQEECEKKVPMCDQPKDVGLCRAQMFRWFYNKQTHKCEKFVYGGCGGNDNNFETVEECERMCPDLVICPWVNVNGESLEPCSRSEACGNATCPGNADARCVVDPCTCGPTFVDKQGHSVSCVAETLEESTSLRTMSVAQHTRCESMLKRHLIVGDSSYVPQCDDKGEFYPVQCTQEKGSKAQCWCVDEAGMQLPSSSTFPKGTKTCKLVKVQKVEVTLAFKHKDASYSETMVSAIKRLIEQFLMDMKAEYDKLSVIVRPD

							ATHVMFTLLSDNKVQLAFQLEEMVRMQGIFIILPEAEMPVDYRASHFYHILDSKAEVQEEDNSVIANPISSVKLEKERGWATFLSFTVIGVFGFFILIYVVPLVIHRRRQKKGEEEPTRSSLVGMVFNNITYGVVGKKAADKNSSASDIPKEVIVQTVEAEYETL
7	U-theraphotoxin-Pv22a	Pv_sp_2566.t1	Cysteine rich secretory protein type 1	Cysteine-rich venom protein	16	179	WAEECQILTYDGILGKYVEDYGSCGQNIFVSNEKVPWTFVGEAWFAERYDFSYGSYNNSVSEVGHYTQMVWNGSHRLGCGFHYCAKDVKKPFYNYVCNYCPMGNDPKRLGMPYSSGKPCSECIKHCKYKKLCTNTCPYADLWVNCAQLNVTWNDWLCSNPEHQRHRGCRATCLCPGKVI
7	U-theraphotoxin-Pv36a	Pv_sp_3922.t1	Cysteine rich secretory protein type 3	GLIPR1-like protein 1	32	331	LHNMYRSNVTPPAADMNFMEWDEYLAKAAQDWANGCRFMHAFPPYVEPGSMGQNLYMGHDPTGVRALLLWNEEYIYYDIKTLDCVPGKQCGHYTQLAWGDSKSVGCGMKRCAIRRFLVVCHYSPIGNIAGYAPYNIGKPCSQCRTGAGGLCANNMCVTKELCERSPEKCEYDCPLKCRNCGTLDKESCKCTCSDGWDSLDCSRSCKNAHDRCGKNPGWPSVTSCPMNNHAVADTYCRKMCGKCRGVQWSDPEKLCCNGTVCANGYILSEKQNSCTCDLLCPGPLCYITPDYDVLETTSADVGSSHHTKAVYGSFTLVALINFLLIPSVHGL
8	U-theraphotoxin-Pv24a	Pv_sp_6678.t1	Tachylectin 5A (IPR002181)	Fibrinogen C domain-containing protein 1	8	262	TTKSPCEKSAKAETYLDTAADMISRVKENLPKDGGVCKCPRKPMDCEQLLNCGHTQSKVYKVWPKNRIIDGGISVFCDMETDGGGWTVVQRRGDYKRASDFFNKKWKEYKTGFGDKERDFWLGNDNIYALTNQRKYYLKIDLQDSENNTRYAFYDQFWIDNESQNYKLHVYDYFGDAGDSLAPTHGGQMFSTTDRDNDKSSTQHCAAKYKGGWWYNECFEANLNGLYDMNGNSVGEEGIKWNSWKSDESLTFTEMKLRPVDY
8	U-theraphotoxin-Pv42a	Pv_sp_2022.t1	Tachylectin 5A (IPR002181)	Ficolin-2	8	363	QFSVLGNELRTEMTSLRIVASSSGSRCGSVDFTDDDDDLQETGIQGRASPRSGRRVSGNSLAGGSASSVEDTLDVLVQSLGNSTREIREEIHQGIQDIDSKLTSISRLSCSEGLETSESVVSARKIPPPQQDVAAKSEVCSRVARNVTSPQSCADLRRGGATCDGVYVIFPQNVRAVRTLCDMTTEGGGWTVLMQRGDFNNKMSSFHKNWASYKNGFGDVEGEFWIGNDVIHLLSAQDENILRVNLEAFDDDVLNLTYSSFSVADENDNYKLRLGVTVGTPSPASNSLRYHSNHPFSTFDKKNDNFEQNCAATYKGGWWFNGCYFGFLTGEYFRPGEKRENWQGILWYDWKGNASLKGAQMKI
9	U-theraphotoxin-Pv25a	Pv_so_5176.t1	Venom Serine protease (IPR001314;IPR001254)	Transmembrane protease serine 9	10	220	NATICTASVLSPVTCLTAAHCVADDNGNAQNPSYVGMVLGTLSADGTGGLIASVAEIIVHPNYNEGGTLQNDIAIVKLSSPVVFTEKIQPVCLGSAPPPFSKCTISGWGKFLHILGVSNVLRYSPHQIVYFDFICKIGYSNFNPTTQLCIEGLGLGSCNGDSGGPLVVQRNNRLEQIGVVSYGRPVICNAPIIGLVVYTEVYAFRDWVLQVSNDPQICIA
9	U-theraphotoxin-Pv29a	Pv_sp_7148.t1	Venom Serine protease (IPR001314;IPR001254)	Coagulation factor IX	10	278	PVCECGSNSKPLQTRVVGGKNAAAGDFPYATGLLASYNIIFGRQVLGTMDYTPYCGGTLITDKHVLTAAHCVRDRTASQIRLDVGEYSIEDKKESRIIRDARSLLKHPKYVPKVFRNDIAIIELAEPVKLNSKLKAAWLPPQDARIASGTPVTVYGWGRLDYEGGRPQVLQMLQMPVVSRSECQEHLVTPITPNMLCAGGEQGKDACIGDSGSGLTTKRKDENVVVGVVSFGRKCALPHIAGVYTKVSGFSDWIYENTRSTECQPCIASLEDFAEDSQ

9	U-theraphotoxin-Pv37a	Pv_sp_1715.t1	Venom Serine protease (IPR001314;IPR001254)	Serine protease hepsin	14	334	DGCLTVDNGAGECTVFMECRQAVVELKTKFPTICTWDGDVPIVCCPKEKQE<br>DIDDDMLPRTLRLADCGRRAAVAKRPTIAGGVEAQPGAWPWMAGIYIRNFG<br>RDVFLCGSAIVSDKYVITAAHCFGQRGGSVIQTARYAIFVGSLKVKEGTLHTLS<br>SITIHPQYKPREHYNDLAVIEVRERMTFSTYVRPICLPSPGEFQDIDGEDVTVIG<br>WGDQDFGGVQASVLREVTVQVIANKDCNDAYTKLRGSSIPEGITNKFICAGV<br>PEGGKDACQKDSGGPLMVARDGVWSLVGIVSFGYQCAQAGYPGVYTRITEYI<br>DWLDESINVEDSSPVPVAQ
9	U-theraphotoxin-Pv39a	Pv_sp_4039.t1	Venom Serine protease (IPR001314;IPR001254)	Coagulation factor XI	12	341	TPCPLANLQCCPFPSNETESLEDDGYEHSPVLYLDDDDNDNQSGMENPRGTT<br>VPYSQTQQTVTPSSVEEVILFEPTQNLACGQAPYEPFIAGGVESDPFKWPWMA<br>SIFQRFTVARPHKFLCGGSLINTRYILTAAHCCVAGANNIPRPASAFLVRMGS<br>NHLDQGGEDFSVSKVIVHSNFSYSGQYNDIALLRLGTKVAYTDRVAPVCLPD<br>VSLAEQDLEGLLATVVGWGATSVGGEHQQTLHEVEIPVVPTEDCVLAYSRIKS<br>AAFLARGSTHVLCAGLKEGGKDSCKSDSGGPLMMQSDGIRWTIVGIVSFGHR<br>CAEPGFPGVYTKITHYLEWIHSNAIE
9	U-theraphotoxin-Pv40b	Pv_sp_6027.t1	Venom Serine protease (IPR001314;IPR001254)	Serine protease 27	14	344	SVIYPTDPPFQEVCDEANTICKEAASCPDFNTQTEICGWNGSRPMVCCTERRN<br>TGKTEKLHPNTECGIQGGTNRQAVSRISKRQAGLEIEHGEPPIVGGKDVEPFSF<br>PWMGALDKVAYRKNHRFLCGSSLISPRHLLTAAHCFQRGDDKTPQKFSVRL<br>GAHGLKDGTRYSIRTIVIHPGYNFRQNYNDIAVLTLAEEIPPSQKSLPICLPEAE<br>AGRRENLVGRSATVIGWGHTSYGGVNSRVLQMVSIPIVDTGKCNSTYMTIVH<br>RTFPRGITDDFLCAGVEEGGKDSCQNDSGGPLQISDGGRFIQVGIVSFGYGCA<br>EPGFPGVYTRVASYVPWINEVMAKTL
9	U-theraphotoxin-Pv41a	Pv_sp_3053.t1	Venom Serine protease (IPR001314;IPR001254)	Complement C2	10	362	ALFMITDGEPNVGDLGITPRDAASSLKEKNDFEIFTVGIGKGINTDFLTSIASEP<br>PESHVFILKKYSQLKDMISIIRTLPEERPKPGPEKCGYIDESRKPTEYERWPWLA<br>AVYTSFPKADVQQPGHFKMSLCAGALICRDWVLTAASCFHETNRREIVLSLR<br>EVFVVAGEHNILEIDKSQKNYYATDVQTHPDFDPFNPLLNNLALIRLNEAVPL<br>NITFRPICLPRTDLIRPLHEDLATNTSTAGWGRPLKLGFTHGINAATTNFNLRT<br>KPIALAEEGECLRRLKRKAMGNIFCAGVGESDCLGTIGSPIIAQDPNTEIRHILG<br>VLTDKSDCNQGYSQFLELRRYIPWIDGITNSCRLRHQM
9	U-theraphotoxin-Pv40a	Pv_sp_5436.t1	Venom Serine protease (IPR001314;IPR001254)	Enteropeptidase	14	368	SVTFPGGSSNRDGCEDSPKGTVCVVSSRCPGFNRNMKRCGWSNLEPKVCCEE<br>KKLAPVSLPQNLNCGIRPKRNPSGALRKKRETAQDRLWRALQLSKSIESLPLN<br>APEPGLGLRVSKPSVVGGQEVEPLSYPWMAALDKKTYGTNFRFHCGASLISG<br>RHILTAAHCFLKGEEKSPTNFTVRLRAHLKTGGTRYNVRKIEIHPGYNGRENY<br>NDIAILILSREVSITDYVSPICLPQAETSRVRNLTGTTAHVLGWGHTSYGGVDS<br>QVLLEASLPVVDTAQCNAAYKNVASRSFPRGITNYLLCAGYNEGGKDACQN<br>DSGGPLQVLTNNRWFQIGIVSFGYGCAEPGFPGVYTRVSRYVPWIEEVMSKTS
9	U-theraphotoxin-Pv43b	Pv_so_7495.t1	Venom Serine protease (IPR001314;IPR001254)	Proclotting enzyme	14	381	IGSPCVTPDRREGQCVLVTECPVLLRVNNFNRLRRFICGYEDEVPKVCCPEESI<br>GGQPQTPRPPTRRTPPPPPRTPTPTRRTPTPARRTPTPTRRTPSPRPPQPITPPSV<br>PGRSRPDFLPSDCGHSNTTKTRIVGGREAEKGAWPWMAVVYQTKRGGRKSP<br>DCGGALVSRSHIVTAAHCVVGRGIEPLSPSVLSVRLGEHDLYSDDDDMNPVEI<br>NVRSVRRHDRFDPRTYRNDIAVLLLERPVEFNPNIQPICLPYDSLKDEDLTDRS

							GFVSGWGTTSFNGASSNVLREVSFPIVDDEFCRDAFRQDLPITDVYICAGTAD<br>GSKDSCQGDSGGPLMLPGRGGRFYLIGVVSFGKRCAEPGYPGVYTRITKFLDW<br>LTQNLSS
9	U-theraphotoxin-Pv43a	Pv_sp_2944.t1	Venom Serine protease (IPR001314;IPR001254)	Transmembrane protease serine 12	14	429	SIIFPKESSETEEEGPQPCVTPRGRNGHCVIVTRCQLLWNHRRNYSLLRRLNCG<br>YQGEVPKVCCPVHSTPSTQQTTTTITTTTTTPSPVSTGRPYPQRGRRPLAGIPLL<br>PPHEEPGTRGQAALDRSGSVTKKLPSGSSQESIGSNKGVNNSKLATIGRNYDEI<br>ISLLPKECGKNDVPLRRIVGGHESIIGAWPWLAALYLTRRGIKTAECGGALVT<br>AKHVITAAHCTVNSRRGTTFDASQIIVRLGDHDLDSDEDGLTPVEVRVSNIIK<br>HENFVLRTFQNDIAILELVSSVQFHKYVTPLCLPYGVFQGDDLAGRNAFVAG<br>WGTTSFDGPASSKLLEVQLRIWENSDCEGVFRRDVPITNVNLCAGDGDKDAC<br>RGDSGGPLMLPYRDGKFYMVGIVSFGKKCAEPGIPGVYTRITAFLDWIANNIK<br>STL
9	U-theraphotoxin-Pv47a	Pv_sp_3992.t1	Venom Serine protease (IPR001314;IPR001254)	Serine protease 27	14	449	CRYRRTPGTCKRRSECNQPTRRVCRVGLDPIVCCLDRPDEDHKPSIIFPQETPQ<br>QRPRPTTRKPSIIFPQETPAPRPKPSTPRPRPSTPRLRPSTPRPRPSTPRPKPPQAA<br>KPPPPKPAPQKVPDLTFPGCGSRSPSVPDNNARTSARIGGSGGRSSRRRGFWR<br>SKREISASYDAGDLDVNEPAVKKRFKRQMFRPVVVGGIMAKPNSWPWMTAV<br>FKKSTPGSTSRFLCGASLISTLYVISAAHCFDAERGNIDPSKFTVSLGAHNTRDG<br>TEYPVESIKIHPNYQQRQYYNDLSILKTARPVKLTNKVYPVCMPQPTIVSRIVT<br>HQNVTITGWGDTSFGGVGSKVLHEVSFPVVPQRQCNASYAKIASSTFPRGITD<br>NFICAGLEEGGKDACQGDSGGPLVMNTVEDKWVQVGVVSFGYGCAQPGFP<br>GVYTRVEKYTQWLYDNSDLGRQ
9	U-theraphotoxin-Pv49a	Pv_sp_3247.t1	Venom Serine protease (IPR001314;IPR001254)	Serine protease 27	14	475	CSTGEGRAGICVAKRYCREAVASPRQTLTACSGTSGFYCCPVPTARSSEDSSSS<br>DQAPPAAPVRPTPSAVGTSTPRRDIRYPESDSRYRPGSNAEQSDYSSSSSSTHD<br>GKYRYPDKDDSPRETRVKEPDNTNSGSRHYHGEVRLPNRDKPSTKRPIYEDQ<br>DRKHVPNNNSRRPSVQRTTSSLPRRNDRSQEKYTTPTSLIKFPEEYKPPHPPAP<br>ACGQKPYELFIAGGEESRQHEWPWMVAIFRRHSSPKPNTYICGGSLINRRYILT<br>AAHCFIHNYVILPASTFVVRLGAHYLDSGEDYTVANLVVHQNHSGSDFFNDI<br>ALIRLASEVFFTDKIAPICLPTREMSYKTFVDRMATVAGWGSTAFYRIADSRVL<br>QHVSVPVLLNEECSSAYSRVRGAAFLARGTDHIICAGLREGGKDACLGDSGG<br>PLMLKGDDDDSWTVIGIVSLGYKCAEPGYPGVYTRVTHYLSWIYSNMKN
9	U-theraphotoxin-Pv54a	Pv_sp_3640.t1	Venom Serine protease (IPR001314;IPR001254)	Neurotrypsin	28	524	VEVLVNGQWGNVCVDNWQYKEAQVVCRQLGYPLGALEATKRSQYGTGTG<br>NYVLCGLICFGNESSISECGLDIFPCSRCSRGKAAGVVCREYEKTCPEYYFKCQ<br>NDRCVDYGSVCDGFDDCKDGSDEFPSLCKEPEVRLVDGNDKYSGRVEIKFLN<br>IWGTICDDTFDDKDAQVICHSLGFRGPATAHGNAFFGPGNGVIWLDGLHCN<br>GSEAKITRCPRERWALHDCKQYEAAGVTCSSPQGHDLIESMECGIESGDHISP<br>NYEFRRLPGRSSLKLKEINRMTSPMLSSRATALRFPARRRVARMVFSEKITLLA<br>SFPWIADVRYHAQGKSYHACGAAILSEFFIVTAAHCVNKHTKRDYIIRVGEFD<br>QNKKDRFEEDFEVERIIIHEKFNEAVRLNNDIALVKLRQKAKRGIHFSEDVRPI<br>CLPTPETPYLPEIWAAVGGWGITEHSTKSSILRLAYVPLLSPVQCKGFTDYGTN<br>KIRPGMLCAGHIDGGSDACKADSGGPLVTKFNGRSTLLGIVSWGHGCGEA

9	U-theraphotoxin-Pv59a	Pv_sp_2198.t1	Venom Serine protease (IPR001314;IPR001254)	Neurotrypsin	18	655	SCGSGRCPGNRNQDSHPGGNNNKDNPGGRDKDRPAILGPDDWPSSSTGGRP<br>GIGSDYQPDTVGGGGGSDKDRPAILGPDDWPSSSTGGRPGIGSDYQLKPGGE<br>GTYEKDDYVPKPVSGGGGGGRDDYNPKPVTGGGGGGGRDDYNPKPVRGGG<br>GGGARDDYNPKPVRGGGGGGRDDYNPKPVTGGGGGAGGERDDYNPKPVR<br>GGDDYNPKPVRGGNDDYNPKPVRGGNDDYNPKPVRGGDDDYNPKPVRGG<br>DDDYNPKPVRGGNDHNPKPGKEPWSPDTDRHPKPIPESNDNPKDDSYNDGS<br>SKDNIRENIRPGGNCICVPYYQCKDGHIVTDGAGIIDARRKPNNEEELPLDGK<br>FKPPSCGPFHVCCNTPETSTVKPYEHRCGVRNPSGINSRILSPSNAGEADFGE<br>WPWQAAVLKSEGTVNIFQCGGVLIDKYHVLTVAHCVFHLRKYNVFPLKIRLG<br>EWDTQSTSEFLAHEDYKVSKIIIHPEFRNHSLWNDLAILKLEEAVLFAPHIDTV<br>CLAKHDENFAGQNCVVTGWGKDAYKGGTFSNVMKEVAVPVIDNYNCEEM<br>LRKTRLGRFFQLHEGFLCAGGEHGLDSCKGDGGGPLVCYRKDRSYALAGIVS<br>WGIDCGQPGVPGVYVKIQKFLDWISKNTGLQLEDYWPKSY
9	U-theraphotoxin-Pv61a	Pv_sp_435.t1	Venom Serine protease (IPR001314;IPR001254)	Prothrombin	56	993	ILPIGNLDIGLGVNVNGKPEPKPQCDPTDITCTCGIGGRSIFIRVPQCTYAHRW<br>KTKCKPCDKMEPKDVCPKFQNCLQCHLNGGDQCASCPPRKFGNWCENDCF<br>CENGGDCDRSGRCICLPGFAGLRCERRKGCPPPQLVAPPLQENRQPPNNPAT<br>IVYACPSDYVLRGSPAITCQPEGQWSGSPPQCLRKCPVLSAPANGRLEYTSNES<br>VEGTSAITRCNDNFQLVGQSTIHCLPGGVWSSSLPRCKRLSSCSDPGSVAHSTR<br>HIESSNGLQTGPFLEGTQIQYSCLEDYEQMGTDTITCLSDGKWSDERGVCLKV<br>SSISPDCSTRGEEIAEEIGVPVRILCPPECANGAPRLWGTSIYKKESAVCPAAVH<br>SGIVTNTGGLVDVISNGEYDHFVGSNLNNVRSQSSDDRADSFRFNKVPPLVIS<br>KDKEGCPKNLLRLQKSCVYASNNRRNKAEAEAICKNLGLHLSFADSDEERK<br>NLFAVLSEKGITKIWTEKAENHRADGTAPNSCETASISDANTITEEPADCQMR<br>TNFVCAYGSNIRPLASCQDQGTLTNGQPKAVGKIGDVFYVGSSIEYTCDTLHY<br>MKGERIVYCTSNGTWSAPKPTCTRLNTCEAPQVPIGGGVKYFPPLSNGGTQRS<br>GDVRTAIQASRMARLPAGLAAPVPASLSGSSPPQDEEEIENIELPPGVYRVGTR<br>AMYDCESRYYQLVGSRTRRCESRGNWSGRPPTCIPVCGRSDSPRSPFIVNGNA<br>SDIGQWPWQTAIARWLPDYSRWFVLCGASLLNERWVITAAHCVTYAASTLPI<br>DPDRFQVYVGKYHREDSKDDEHVQVKKVHEIHIHPDYDPNLFDADIALLQLE<br>TAVQLNSRVQPVCLPSEQTSRENLNEGRQGVVTGWGMNENETYSETLQQAV<br>LPVVSHGTCEKGYRDSKLPLTITDNMYCAGFAQGKTDACSGDSGGPMVFLDE<br>SAKERRWILEGIVSWGSPSGCGHANQYGGFTTVSKFLDWIHLFF
10	U-theraphotoxin-Pv28a	Pv_sp_8942.t1	Protein disulfide-isomerase (IPR005792)	DnaJ homolog subfamily C member 10	6	258	EEEKLLVLFTEECCSCTECVETEVLLGAMSADIENTFNLPIVKLKKTDMRKSYG<br>VKTVPSLVFIRNGKPVQFDGQFEKEALFIWIQDNLDPATTHLDDHNFEHLTQ<br>AATGATTGDWLVAFYSTACFKESNILTNMEGAGSHLRNRVNVALVDTEKSP<br>ELVQRFKIKHCPEIIFFRLGKMYRFSLSESSALALKRFAEGFYKNSKSETVPVPKS<br>PFDKLIEQLFESTRQYMFELTVFMITLTLVVMFIIILSVLKKGQE
11	U-theraphotoxin-Pv30a	Pv_tr_4119.t1	Protein disulfide-isomerase (IPR005792)	Protein disulfide-isomerase A5	6	283	KLTVQSMVNFLKDPSGDMPWEEDEHAQDIVHIPDANALGKVLHKEKVPIML<br>MFYAPWCGFCKRLKPEYEAAATELKGHSILAAMDVNKPENSIVRKHFNITGF<br>PTLLFFEGGNLKHKYDGENNKDSIVSFMRNPTKQPEKPKEPEWADTPSDVVH

							LTDATFMSEIQSEPSVLVMFYAPWCGHCKKMKPEYVTVAARLKQQNIPGML AAVDATKEKTLADQYKINGYPTLKYFRNGEFQFDVSLRTADQIEEFMMDPKE PPPPPPPEKEACSSHVLCTMVWPLQ
11	U-theraphotoxin-Pv33a	Pv_so_7333.t1	Protein disulfide-isomerase (IPR005792)	DnaJ homolog subfamily C member 16	8	296	YDLHGEEGLKDDFGSGWQGSFRSWNYYYESFGIYDDDPEIITLSRSDFEQSVIG ARDTWFINFYSPQCSHCHHLAPAWRALARELEGVIRIGAVNCEEDWMLCRQ QNIHSYPSLMMYPQQTKYRGSRDTDSMVDYVLQQLPDVVIEITGHNFQELVS REEYKVHPWLISVCKEDEECLSSEALRKLSLILEGLVNVAKAEYGLDRAVCDK LDCRSLITFYSNLSLPVETVAVHQIKETEVKPVLNEVLLLLPGATEMTEEMFQN VRKGDSLSYKPWLIHFTKSSLKESDGNMDTEL
11	U-theraphotoxin-Pv44a	Pv_tr_7700.t1	Protein disulfide-isomerase (IPR005792)	Protein disulfide-isomerase	4	434	TEEHVLVLNKDNFDEAVKDKNVLVEFYAPWCGHCKALAPEYAKAAEKLEE EKSEIKLAKVDATEETELAEKHGVRGYPTIKFFRDGKILEYTGGRTSDDIIRWLK KKTGPPASDLSSADGAKSFIEGSEVVVVGFFKDQDSKDAKVFKDVAAEMDDY PFGITSDDVVYNELKASKDGVILFKKFDELRNEYDGELSDEELKKFVKSNSLPL VVEFSHETAQKIFGGEIKAHNLLFISKESADYSEKVETFKKVAKDFKNKVLFVT INTEEEDHERIMEFFGLKKEEAPTMRLIKLEEEMTKYKPETAGISEDEIRNFVQG VVDGKVKQHLLSEDIPEDWNKEPVKVLVGKNFDEIAFDKSKNVLVEFYAPW CGHCKQLAPIYDQLGEKYKSSADVVIAKMDATANELEHTKINSFPTIKLYRKD TNEVIDYNGE
11	U-theraphotoxin-Pv46b	Pv_sp_3707.t1	Protein disulfide-isomerase (IPR005792)	Protein disulfide-isomerase	6	448	VHEHDTILVEFFAPWCGHCKRLAPEYDQAAAALKKADPPVPLAKVDCTSDS GKDTCSKYGVSGYPTLKIFRGGEFSSEYNGPREADGIIKYMKSQVGPSSKELKS VEDAETFLSKDDVVIVGFFKDTSSKLKEEFLKVADKMRESSTFGHTVEPEVLSK YEFSEQIVLFRPKKYWSKFEPQAVQYTGSADKGQIQQFVKETYHGLVGHRTH DNHEDFKAPLIVAYYDVDYVKNVKGTNYWRNRIMKVAQNYKDKVNFAISN KDKFSAEVEDYGLTTKGDKPIVAARNTKLQKFNMKEEFSIENFEKFVQDFVD GKLEPHLKSEPVPETNDGPVKVAVAQNFEELVTENTKDVLIEFYAPWCGHCK KLAPTYEELGTRMADEDVEIVKMDATANDVPAPFEVHGFPSLYWLPKNQKS SPQRYEGGRELDDFIKYISKHATDELNDYDRK
11	U-theraphotoxin-Pv48a	Pv_sp_3723.t1	Protein disulfide-isomerase (IPR005792)	Calsequestrin-2	8	467	VCHLTAENYTTVLSAAEVAVVVFTAPQPTKQPTVCPTELDNFAEVSAQVLRK KNIIVCEASADLLTSQQTAPVPQVNAGDVYIYKKGQGVPYYGRRSTPALLSFL FKVNGTQVNVITGKIDKIAFDAVQGTKIVGFFMQGTADYNAFEEAAAKLSPS VAFYVAFDRVVAKHLKLETVGQIHLIKPLEKTPIPCPQNPASAADIEAFVGSQ KGAILTKMNEHNLYDPQLLDPSRTLVLAIGEEASSFGGYFYHLVTKLVRNNT NNTEFEKLNIVWIEPQIFPTIHLMMSELETTLGIPNKLPAFGTVNITSMQSAWL NTALLNTTSDKTSDEANLKILQDFLSSVINNTIVPVKIGSQSFVQMPASQVVAE GSDVLLECVIENLVGDCLWLRNGQNIGFNLARFTQYSWRGDQTAGDCSLQIT GIQKGRDDGEWVCEVTGDAENPTVTSSPAKIAISGAADTLAKSEL
12	U-theraphotoxin-Pv38a	Pv_sp_4280.t1	Cystatin (IPR027214)	Cysteine proteinase	8	339	SLSQMYKLDYFNCDLDSSKGQRSVSDKEHIERGMFADFVAKFSKVYESEEEEE LRFRIFQENLEKIKLHNDLERGTAKYGVTKFADLTATEFRKYALGFRPDLLDE DNPLPLASTPKDPIPTSFDWRTKGIVTEVKDQGQCGSCWAFSTTGNIEGQWAI KKTKLVSLSEQELVDCDKVDEGCNGGLPSNAYKEIIRLGGLEGEKDYPYEGED

							EKCNLNKTEVRVYINSSLAISQNETEMAAWLVKNGPISIGINANAMQFYYGGISHPWKFLCSPKNLDHGVLIVGYGVHSYPLFKKTLPFWIIKNSWGATWGEQGYYRVYRGDGTCGLNLMATSSVVD
-	U-theraphotoxin-Pv5a	Pv_sp_10092.t1	Huwentoxin-1 family, Lectin subfamily (IPR011696;PF07740;)	Kappa-theraphotoxin-Gr1a	2	26	FCTFLVHAAPAKKRQRFFCVIDSSTQ
-	U-theraphotoxin-Pv6a	Pv_sp_14704.t1	Huwentoxin-1 family, Jztx 36 subfamily (IPR011696;PF07740;)	Kappa-theraphotoxin-Gr2c	6	30	DCRQWKQPCNNQYRCCKGSYCAMVCIQMWG
-	U-theraphotoxin-Pv8a	Pv_sp_8204.t1	Magi-1 superfamily, Ltx4 family (IPR012627;PF08092;)	U1-theraphotoxin-Tal1a	6	37	CLGENVPCDKDRPNCCSKYECLEPTGYGWWYASYYCY
-	U-theraphotoxin-Pv9a	Pv_tr_11074.t1	Huwentoxin-1 family (IPR011696;PF07740;)	U1-nemetoxin-Csp1a	6	37	KCKPYGQKCKTPQDCCSEACSSDNECSNREVVLMLYG
-	U-theraphotoxin-Pv10a	Pv_tr_16283.t1	Plectoxin superfamily (IPR004169;)	U8-agatoxin-Ao1a	8	42	ACVRRGSSCDGKPNDCCPNSSCRCNLWGTNCRCERAGLFQQW
-	U-theraphotoxin-Pv11a	Pv_sp_11289.t1	Cystatin (IPR027214)	Cystatin-C	4	69	QVVAGVNYGITFTVGLTTCKKEEVDYKDINACEFQDNISTYRKCDVIIYRNLDDEYKLTWSGCILTKPE
-	U-theraphotoxin-Pv14a	Pv_tr_15647.t1	Peptidylglycine alpha-amidating monooxygenase (IPR000720)	Peptidyl-glycine alpha-amidating monooxygenase	4	107	LPLLMPDVQPMQKETYLCTAYKMPRSDYEYIVEFEPNATMHTAHHILIYGCSLPGRWERDSPRLVWDCGEMVGVHRGFISGPTCSSGSQIIYAWAKDAPPLKLPEVS
-	U-theraphotoxin-Pv21a	Pv_tr_14111.t1	Leucine rich peptide (IPR032675)	Insulin-like growth factor-binding protein complex acid labile subunit	6	164	LAKNNLTQISAGDFDSYPHLGLLVLDDNCVSEIENDALGRLEYLMRLWLNGNCLSKVPPNLPPSLLALYMEENRLTELTSYSFKGLVKLEQLFLQRNDIRYLEICAFCDLVSLQSLDLQANQIENLTAGVFANLTNLQTLDLSQNILKLLDSRCFEAPCSYTEL

-	U-theraphotoxin-Pv26a	Pv_so_6369.t1	Protein disulfide-isomerase (IPR005792)	Thioredoxin domain-containing protein 15	8	231	NEDYHLPSSESVTMESDAKSQENGTVTEGNVTLTNATSGSENKTSVKVECLLGELEEGEIPAVHLINGTMLIKIFSAPANNSTAGECSIVMFYSPYCVFSARAAPHFNALARVFPGVKCYAVDAMTNGNLHLRYGLVAVPNVMLFHKGKPIARFNETHLNLERLVTFIEKYTGLQYNGTLNVTSADMSGPVPSTLSKKVDYVLILAWIFTICCMCYGFSKSTLCHKIIESV
-	U-theraphotoxin-Pv27a	Pv_sp_3913.t1	Insecticidal toxin ABC family, Calisoga subfamily (IPR012626;PF08091;)	Disintegrin and metalloproteinase domain-containing protein unc-71	12	255	YIELALILDQAFFEMHNSPPKEVIGNAIQMINCADLHYHSLNASISLVHIELWTEDVIHVEPNIEQNLQNIQEYLEQQYDRRSMDAIHMLSGAAFNDSVSGAAETDSICTVKAVGITKVGNIHQTHVTSHVITHMLGHNLGMYHDHDCCTCPYKSGCHMLNHIQSVQPFHFSSCSIEDYFRTLRKGYGVCLFNMPMLRESICGNGILEKGEECDCGTSEVGMDIATRETAQLLTISVHSCGEVALKQQIQFVTRG
-	U-theraphotoxin-Pv31a	Pv_tr_2015.t1	Thyroglobulin-like protein	U35-theraphotoxin-Cg1a	18	287	ADALEQMSGSSSGDSSHQEKQCRDLRENFDCMLWQTSMCLKREEQDSNSDVIWKTWRYLGGFCEKYINWWANPCFQRDDIKRCESRLPSKSNSLDGQSCRMFVNFRDCVTSIASNSCSNSDRSLLGSYFTEKGKQKAWRCPKDSDMTSLPAASAQQTDRRYPDMYGDSGDSSCLVNARTELRDCQDRFQNAQSEAMRRDDSDMRNHQLCCGLVAYKECLEGAKEKCGPSDTGRIDNIYKTAESQVKVDCTETAESCKGNGASSIIPTNAAAIYFLLYLGYFFSVKTL
-	U-theraphotoxin-Pv45a	Pv_sp_2844.t1	Leucine rich peptide (IPR032675)	Leucine-rich repeat protein soc-2 homolog	6	442	APAGCPPADLISPCQCRSFVSAQVKCENVYDLEIVRSALSHRFPHGLRTLHISNSNILVLPDHAFTNISLTRLLIDGSNIRQIDEDAFAGQEDLLFLSLQNDRLFDVPISALSRLTQLRALILSDNLLIFLGQDAFADLDHLINLVLRNNRISRIERGVFPRNLDTLSLSGNLLTTLNRTLIGLSHLSWLFLFDNRLTSLKGELNGLEKLELLSLSNNQIPDLEDSLGDLHSLEILDVSYNNLHRVGNSLRSLSSLKKLDLSFNYFTELESDSFSSLSQLTQLDLSGNQLMTVSLSLNYLGSLHRLNLSANDLTSLEYEDLKGMSRLRELDLSHNKLKNLDGTFGFQLSELTVLKLQDNQLKRLHRSLKNLRDLKVLDVRDNQLTTLHTSQMKHNKQLEILEIGGNPLSCDDDHLHDVLKDLKTRMVKVTGQPFCYLDENTL
-	U-theraphotoxin-Pv57a	Pv_sp_2795.t1	Peptidylglycine alpha-amidating monooxygenase (IPR000720)	Dopamine beta-hydroxylase	14	535	VPDVYWVAVGFSNRGLVTNADFCFLWTDKRGKNQLQDVWTDEAGYVNVDDHNDCELLNLKRRGHVTRLAWSRKFDTCDPQDYYIEDGTTHVVYAVGKGPIRRLEGIRIVNEKHGFQRVHLLKNLAPAPVFPDDTQTITINNDKVHVPALETTYWCSLHLLPKGFENKKHIIQYAASIQEGNEPVVHHMEVFHCEVSPEQKLPPWNGPCHSDEKPTVLEACKRVLAAWAMGALPFYYPEEAGLPIGGPDFSRYVMLEVHYNNPELKADWIDSSGITLWYTPTLREFDAGVMELGLEYTDKMAIPAHQESFTLTGYCVSECTRAGLPPEGIVIFGSQLHTHLTGIVVYTKHIRGGQELPELNRDNHYSTHFQEIRFLKRRVRVWPGDALLTTCHYSTMDRQNITLGGFAISDEMCVNYVHYFPKTDLEVCKSSIDSQVLNSYFRYMNQYNDEPTSEDKGISDNYNSIHWSQNNGDFLHQLYYNAPLSMQCNKSSGDRFPGYWEGVPRTEVLYPLPPPKKKCFDKVRNNEDLISAVES

Table S3. Summary of the mature toxins from the merge transcriptome of *Pamphobeteus verdolaga* obtained with the BLAST (Arachnoserver + ToxProt) and HMM (hmmcompete) prediction strategies with odd cysteine number.

Cluster	Given name	sequence_id	HMM family_name	Blast2GO annotation	Cys	Length	Proposed mature sequence
3	U-theraphotoxin-Pv13a (Fragment)	Pv_sp_11560.t1	Thyroglobulin-like protein	Thyroglobulin	11	105	WMLPKCQDDGTFTPMQCYPGTTACMCVAADGSPLTLPGFVNVTACDCFTE YYKMWLKDEHSPVLPRCADDGTFKPLQCNKAEGVCWCVDKTGRVLTKPSK DTKSC
5	U-theraphotoxin-Pv19a (Fragment)	Pv_sp_11018.t1	Angiotensin-converting Enzyme (IPR033591)	Acetylcholinesterase	3	134	HSHSFHDRDFFPVVTTSTGDIRGKQVNLDGKKVTAFLGIPYAEPPVGDQRFKR SVPKQSWSGVYDATKKPPPCMQYSTGDFQWTTNSTPSEDCLYLNVWVPGKM NFCELESKSKPVLLWIYGGGFLRVLNLRNT
6	U-theraphotoxin-Pv23a (Fragment)	Pv_sp_7347.t1	Venom Kunitz-type family, Long Kunitz domain containing protein (IPR002223;IPR020901;PF00014;)	Tissue factor pathway inhibitor	19	200	TCPGDLYIGDVCSLPKKVGPCRAAMPRYYFNKETGRCETFTYGGCSGNYNNF ETKEQCNSYCFQDKPRYYPRDIEICDLDPDRGNCDSSLERYYFNRNTWKCEKF TYSGCGGNRNNFVYLFECERSCPGDIDLGDICSLGLEPGIPGTNISCLALIPRYY FNKGSGNCEEFTFGGCGGNYNNFKTMEECQRFCTMGPPPS
8	U-theraphotoxin-Pv24c (Fragment)	Pv_tr_12866.t1	Tachylectin 5A (IPR002181)	Angiopoietin-1	5	215	NDTTREKECVLSQVDMHLSIAAEFVERARKNVPNDLKGVCKTKICPGEKPRD CSDIYKNGHHEDGSYEIFPLSRVMHRPLTVYCDMTKNGGGWTVIQRRGDFGR STDYFFQGWDSYKIGFGDVMKDFWIGNDNIFALTNQRNNQLRFDLTDWDG NTRYATYDEFWISDDIHNYYLHVQGYDGTAGDSFGRHNGHNFTTKDRDND MRSSTNWVSTA
8	U-theraphotoxin-Pv24b (Fragment)	Pv_sp_8847.t1	Tachylectin 5A (IPR002181)	Fibrinogen-like protein 1	7	257	LNLASKMLETARTELRNDMKQLCQSNSCTGNFGCNKWRSSHAPGKPIDCSDI YNDGYHTSGIYQIWPQSKISNGPLVVYCDMEDGGGWTVIQRRGEFGNSDDYF YLGWNDYKTSFGHLLREFWIGNDNLFSLTNQRSYQLKVDLTDWEENSKYAIY QEFWIDDEVHNYTLQIKGYNGTARDSMRRHNGNKFSTKDKDNDPWVGGN CAEKHKGAWWYVTCRDSSLNGLYLRANSATSDNGVRWYDSKILYHSLKDSV M
8	U-theraphotoxin-Pv32a (Fragment)	Pv_sp_2289.t1	Tachylectin 5A (IPR002181)	Angiopoietin-1	5	288	MADALQVGINTLRANLTDLTNLTRTVYDHTKNSLVTKTYFQSSLQPLMTAKE ENPQFIGCMMQSQEPDERLPRDCKDVQELVENKTGIYRIQPKYATRPIFVYCD METEGGGWTVIQRRRDGSVDFLREWSDYKYGFGNVGSEFWLGNENLYLITH QALYELRVDLYDFDDGHAYSKYDGFAVGSEKEKFMLKVLGRLTGGDAGDG MTYHASIPFSTTDMDNDRWEGGNCAEDHTGGWWYNQCDASNLNGQYLA GLTPQEYKGVYWHEWQGPNYSLMGTQMMIRPMKH
8	U-theraphotoxin	Pv_sp_798.t1	Tachylectin 5A (IPR002181)	Fibrinogen C domain-	13	517	IGGGCPINDLKFFLTKKIDSLAASTALLSECLECDRDSLKDVLHTSVRNVMEN VSKRLHLCTTSDLERTVGKILDSKLKTTTDTLQEDISFKLGKVGHQLEDVKQEC GKLNDEEASPSIRRDSHTGVHDIAYSHDTPQELRSGGKHGGDDSGIPKETPGR

	-Pv53a (Fragment)		containing protein 1				IFRKLWRKMTEPINKVGEKIEALAHVVETSNQKHHNETVSRLTRELSSKDKDS EPCTKQLETVAESTKETAQRLGYIESRQLTVQNMCSKILVEVERMRNTVETGK IGIGVGTRLGVTETLDGAAKPGYEVAKSCAELQEQGIVTDGVYNIKPNGAAA EFLAYCDLTTEGGGWTVIQRRGDYGEHLRQNFTQGWDAYKRGFGDFQREF WLGNEKISLLSTQEDVKLRVELEDFDGNTAYAEYSRFRVADESQQFLLSVGEY QGNATDSLSLHDGKMFSTEDRDNDEVSACCNCADTFKGGWWYYRCFEANL NGPYQTNPTDNGYFLGIIWERWKGDYSLKSSEMKIRPLSFEDVRDP
9	U-theraphotoxin -Pv35a (Fragment)	Pv_sp_6381.t1	Venom Serine protease (IPR001314;IPR00 1254)	Enteropeptidas e	11	321	VCCDLPLVLGENLLRTIEEEKARNRECGTQFPTGYNTSPTEERFQNIYRDIPPID RDDQYKYRPPLDFTNYSDFGILVAGGEPATEGQYPWMIAIFKGPTFWCGGTLI DRSHILSAAHCFVNSRDELNPSDYTARVGSIRRNNTVPFKISKIVPHEDYDPRK HYNDIAMLTLECEILTPYVGHICLPDIQLASRDLTGQNVTVLGWGDLYYGGP QSRILQRVTVPVVSNDQCNTVYQSMARNSIPQGLTSDFICAGREEGGKDACQ YDSGGPLMHWSERDYLWTLVGVVSFGYNCAVPGYPGVYTRVSSHMDWITRQ ANEV
10	U-theraphotoxin -Pv60a (Fragment)	Pv_sp_59.t1	Protein disulfide-isomerase (IPR005792)	Protein disulfide-isomerase A5	15	684	EMLFKLIEDQDFLAVFFYAENDKESEEAAHQLEHIDDDCGEFAIHLVKSSDKI MAKKYGIRNPPGLVLFRNGQHIRYGGDLKDQEEVLEWLINPENVATSDIVEK VNSKMFERLLQRFDYIAVFFYSKIGCKMCNKVLDELEKIDDEAEADGVHIVQI EDHALAKKYGVFAYPALLFFRGEENEPIIYAGDIRSGDRMLDWLLTQKDPSTD YIEEMEGLPLQNLIDTADHLAVFFYDKHLCSNCDDDRKDAMECEDCQIILDE LENIDDDTDRHGIQFIKTNDLGLAKKYGIKLTPALVYYEQQVPSIYEGDLAAE EEVLQWLIQQKSEDTIETVNKEMLEKLIAETQYLAVFFYKAHCRACDVALQEL ENIDDDTDLYGIYVVRIQDLQVAKRYGIKTFPALVYFRNGNPLIYDGDLKNEE GVLEWLIDDDNRELPDEIETVNLRMLNKLVEESPFLAVFFYDLNCYECERVLM ELENIDDECDIFGIDLVKIFDPEAARQYNVHSVPAVGFFRKQVPMFYDGDLFD EEKLLKWLTSNDVFEIKDEIEEVNRKMLEKLLEDNEFVAVYFYDNNCPKCDE ALQELERIDDEADDLEIMFVKIKDPRYSRKFGIAQLPSLVYFRKKFPSIYRGNLL EEDEVLEWLRKNRYRHPELNLFMYGLGALSLSFILYTVFLVFCFNKNKE
11	U-theraphotoxin -Pv46a (Fragment)	Pv_sp_2488.t1	Protein disulfide-isomerase (IPR005792)	Protein disulfide-isomerase	5	477	AMGIVDYMKQLADPNWKPPAEAVITLTSENFTDAVNAADIILVEFYAPWCG HCKNLAPEYERAARVLKDLPTPIILAKVDATVEKELGDKYEATGYPTLLIFRK GKKYRYEGPRDERGIIFYMKDQAKPVSREVNSYKSLKNSMSKTDAVIVGFFNS QFENLYEQYVESADFLRGKLQFFHTFDPQIAKQFGVTTSKIVLYQPEIYSSAYEP SKYEFTDIDGTTDDIKMFYRDHILPLVGERSPKSRWQYQDKYPLVVVYFDVNF SFEHRVQTQLVRKEVAKVAKDFKGQITFAVSNEEDYQDELAALSLDDSGEDV NAAYFASEKARYRMEPVEDFSADELREFVENVQAGNIRRHIKSQPQPKENKG PVISVVGSTFEELVTKSNKDVLLEFYAPWCGFCKKFEPTYKKLGKMFAENDKV IICKIDAAANDYPEQFEVRGFPTVYYIPASDKENAIHYEGERDLNALADFVNN QL
12	U-theraphotoxin	Pv_sp_3295.t1	Latrotoxin superfamily, Alpha latrotoxin	Cathepsin K	11	476	EDLGNYGVSYKVAYMPDKLGNPIHTCFEVDGNENNTVVIQSILPDLTNFTFV KSVACTSQNGIVTSNTDCELWTYEDSYGNKVNRYYMYIRKSDRSPVHYIMKG YNTLLGSHYDKYELVYTGYEPGNVSDSDFEVHTAGACRSFPGPGVQEMVLN

	-Pv50a (Fragment)		family (IPR002110;IPR020683;IPR013829;PF00023)				NPMQEFINKVDGHTHAAFDDFTSLHEKKYSSQKEQELRKTIFRQNYRFVNSMNRAGLSYYLKINHLADYTDQEIHRIRGRLPSKGYNGGKPFPKEEFSNDLPDNLDWRLYGAVTQVKDQAVCGSCWSFGTTGTIEGAYFLKTSNLVRLSQQQLIDCSWNFQNNGCDGGEDFRAYQYIMDAGGLASEDDYGSYLGVDGKCHDKNVTKTAKISGYVNVTSGDSLALRQAIAKKGPISVSIDASHKSFSFYSHGVYYDSECKNGPDDLDHSVLAVGYGVMNDEPYWIVKNSWSTYWGNDGYVLMSQKDNNCGVETSPTYVIMAD
-	U-theraphotoxin-Pv15a (Fragment)	Pv_sp_8973.t1	Cystatin (IPR027214)	Cystatin	7	108	NVEDDDVKRIANFVVSENNKQSDSPYCEKLVEVINASTQIVAGINYRITFDTAPTSCLKNDQNCSEMTDCSVLECAPHKRCVANIWSKPWLNSTRIENIEYTERSNSC
-	U-theraphotoxin-Pv16a (Fragment)	Pv_so_1217.t1	Leucine rich peptide (IPR032675)	Zinc finger protein 225	7	110	LAETPAKAGCPRKEDIHPCECLEIPRYPQEGDVQVTETVAFCKTIRNVQVLQNAMKGMQGHTVDFMVLDSCKLPPFPNGLFYNINIKWMEVINSTIQVNEGFFKCASNCW
-	U-theraphotoxin-Pv34a (Fragment)	Pv_so_6686.t1	Leucine rich peptide (IPR032675)	Leucine-rich repeats and immunoglobulin-like domains protein 3	13	308	QASGSNPCSCYYKNSCGCYCPEPITWEQFQLLPANFRSCERFTLALRGGTFYSFPPDYFSRVGSVRDFALDVGDSKFTDLLDSPSPVKGVNFDNAAFLRFNNVTVTNSWNWGAFRDLTPTLPLSYCEIQVLYSTVQSLSADFGRICQGSVTVVNVMYSKMATMDDGVFTNFRKLNEVDLSGNKLQSMRRSYFSMPANELETINLSYNNIRSLPNNMFTEMPSLQKVDLKGNPITTLDETTFRSIFPYVEIIGIEGFPLNCDCRLRWLKEPGQQCGKNLYGLAGAVCNDPTRLRGRSFFNDTLPRDLVC
-	U-theraphotoxin-Pv58a (Fragment)	Pv_tr_11685.t1	Angiotensin-converting Enzyme (IPR033591)	Angiotensin-converting enzyme	11	590	VAAYKFLHENDKVASEMCTKSAIAQWNYASNLTEENKQVMLDTQVLEADFRKETWKNATKFAWKSFKEEPEIYRWFKSLSILGNAALPENKLNQLNKLVADMEDIYSKGKVCKPQSPKGKPCEWSLEPDLTEMMSKSRDYKLLKYIWKSWRDVTGKTVKNDFLKYIELSNEAAKLNGFPDAGVLWRESYDSSTFEDDLEELWETLRPFYQQLHAYVRRKLIHLYTTCNVKNDGPIPAHILGNMWAQTWENIMNVVDPFPDKAEIDVTSKMKEKNMTILEMFQISEEFFTSVGLTPMTPEFWNRSIIEKPQDREIVCHASAWDFCDAKDQRIKMCTKVNMEDLITVHHEMGHIEYYLQYAQQPTVFRDGANPGFHEAVGDVLALSVATPDHLKKIGLLDEVSNDKDGEIKFLMHMALEKLAFLPFGYLIDQWRWKVFSGEIKPEEMNRKWWELRLKYQGVCPPVERTEDELDAAAKYHVISSVPYIRYFVSTVIQFQFHKALCDAAEYRGPLHKCDIYQNKKAGEVLSTVLSQGSSVPWTEAMGIMTGGVTQKMDASAILEYFDPLTKWLQEKNKNEFIGWKSDDPMLCPS

Table S4. Summary of the toxin precursors with signal peptide associated sequences, from the merge transcriptome of *Pamphobeteus verdolaga* obtained with the BLAST (Arachnoserver + ToxProt) and HMM (hmmcompete) prediction strategies.

sequence_id	HMM family_name	Blast2GO annotation
Pv_so_352.t1	Huwentoxin-1 family, Jztx 60 subfamily (IPR011696;PF07740;)	NA
Pv_sp_13202.t1	Huwentoxin-1 family, Jztx 36 subfamily (IPR011696;PF07740;)	Kappa-theraphotoxin-Gr1a
Pv_sp_15084.t1	Huwentoxin-1 family, Lectin subfamily (IPR011696;PF07740;)	Kappa-theraphotoxin-Gr1a
Pv_sp_12559.t1	Huwentoxin-1 family, Lectin subfamily (IPR011696;PF07740;)	Kappa-theraphotoxin-Gr1a
Pv_so_347.t1	Venom Kunitz-type family, Native Kunitz toxin subfamily (IPR002223;IPR020901;PF00014;)	Kunitz-type kappaPI-theraphotoxin-Hs1b
Pv_so_222.t1	Spider WAP family, Spider wap 1 family (IPR008197;)	U15-lycotoxin-Ls1d
Pv_sp_14336.t1	Spider WAP family, Spider wap 1 family (IPR008197;)	U14-lycotoxin-Ls1b
Pv_tr_261.t1	MIT-like CsTx-21	Astakine
Pv_sp_12447.t1	Huwentoxin-2 family, TXP1 subfamily (IPR012625;PF08089;)	Omega-theraphotoxin-Ba1c
Pv_tr_2182.t1	Spider WAP family, Spider wap 1 family (IPR008197;)	U15-lycotoxin-Ls1d
Pv_sp_13299.t1	Huwentoxin-1 family, BsTX5 subfamily (IPR011696;PF07740;)	Toxin-like protein 14
Pv_sp_8246.t1	MIT-like CsTx-21	Astakine
Pv_sp_9798.t1	MIT-like AcTx family (IPR020202;PF17556)	Astakine
Pv_so_3402.t1	Leucine rich peptide (IPR032675)	NA
Pv_sp_13292.t1	MIT-like CsTx-21	U33-theraphotoxin-Cg1c
Pv_sp_9171.t1	Huwentoxin-1 family, Lectin subfamily (IPR011696;PF07740;)	Kappa-theraphotoxin-Gr1a
Pv_sp_11881.t1	MIT-like CsTx-21	NA
Pv_so_464.t1	MIT-like AcTx family (IPR020202;PF17556)	U33-theraphotoxin-Cg1c
Pv_so_5544.t1	MIT-like AcTx family (IPR020202;PF17556)	Astakine
Pv_tr_6792.t1	Spider toxin Tx2 family, Spider Tx2 sb1 (IPR004214;PF02950;)	Hainantoxin-XV-3
Pv_sp_12979.t1	AVIT (prokineticin) family (IPR023569;PF06607;)	U34-theraphotoxin-Cg1a
Pv_sp_9651.t1	MIT-like AcTx family (IPR020202;PF17556)	U34-theraphotoxin-Cg1a
Pv_sp_12678.t1	MIT-like CsTx-21	U1-hexatoxin-Iw1e
Pv_so_1872.t1	MIT-like CsTx-22	U34-theraphotoxin-Cg1a
Pv_sp_11357.t1	MIT-like AcTx family (IPR020202;PF17556)	Colipase
Pv_so_2836.t1	Leucine rich peptide (IPR032675)	Leucine-rich repeat-containing protein 4B
Pv_tr_10028.t1	Venom Kunitz-type family, Long Kunitz domain containing protein (IPR002223;IPR020901;PF00014;)	Tissue factor pathway inhibitor
Pv_tr_10034.t1	Venom Kunitz-type family, Long Kunitz domain containing protein (IPR002223;IPR020901;PF00014;)	Kunitz-type U19-barytoxin-Tl1a
Pv_sp_6867.t1	Venom Serine protease (IPR001314;IPR001254)	Chymotrypsin-like elastase family member 2A
Pv_sp_8677.t1	Tachylectin 5A (IPR002181)	Fibrinogen C domain-containing protein 1
Pv_sp_7161.t1	Tachylectin 5A (IPR002181)	U33-theraphotoxin-Cg1c
Pv_sp_9105.t1	Tachylectin 5A (IPR002181)	Ficolin-2
Pv_sp_8711.t1	Tachylectin 5A (IPR002181)	Fibrinogen C domain-containing protein 1
Pv_so_6999.t1	Leucine rich peptide (IPR032675)	Insulin-like growth factor-binding protein complex acid labile subunit

Pv_so_7124.t1	Leucine rich peptide (IPR032675)	Leucine-rich repeat-containing G-protein coupled receptor 4
Pv_sp_6615.t1	Leucine rich peptide (IPR032675)	Leucine-rich repeat-containing protein 4C
Pv_so_6904.t1	Leucine rich peptide (IPR032675)	Leucine-rich repeat and fibronectin type-III domain-containing protein 5
Pv_sp_5116.t1	Leucine rich peptide (IPR032675)	Slit homolog 2 protein
Pv_tr_4644.t1	Leucine rich peptide (IPR032675)	Leucine-rich repeat and fibronectin type-III domain-containing protein 4
Pv_sp_3739.t1	Cysteine rich secretory protein type 2	CRISP/Allergen/PR-1
Pv_tr_10255.t1	Peptidylglycine alpha-amidating monooxygenase (IPR000720)	Dopamine beta-hydroxylase
Pv_sp_2598.t1	Huwentoxin-1 family, Hntx 3 subfamily (IPR011696;PF07740;)	Serotransferrin
Pv_tr_12500.t1	Latarcin superfamily, Lt7 family (IPR018802;PF10279;)	Neprilysin
Pv_sp_1860.t1	Latarcin superfamily, Lt7 family (IPR018802;PF10279;)	Endothelin-converting enzyme 1
Pv_sp_1652.t1	Latarcin superfamily, Lt7 family (IPR018802;PF10279;)	Neprilysin
Pv_tr_11735.t1	Venom Serine protease (IPR001314;IPR001254)	Vitamin K-dependent protein C
Pv_tr_7613.t1	Leucine rich peptide (IPR032675)	Toll-like receptor 2
Pv_tr_11818.t1	Venom Serine protease (IPR001314;IPR001254)	Complement C2
Pv_sp_270.t1	Tachylectin 5A (IPR002181)	Collagen alpha-2(I) chain
Pv_so_5188.t1	Tachylectin 5A (IPR002181)	Angiopoietin-1
Pv_sp_5783.t1	Venom Serine protease (IPR001314;IPR001254)	Transmembrane protease serine 11B-like protein
Pv_tr_10029.t1	Venom Kunitz-type family, Long Kunitz domain containing protein (IPR002223;IPR020901;PF00014;)	Tissue factor pathway inhibitor
Pv_so_7554.t1	Leucine rich peptide (IPR032675)	TLR4 interactor with leucine rich repeats
Pv_sp_1472.t1	Leucine rich peptide (IPR032675)	Follicle-stimulating hormone receptor
Pv_sp_1726.t1	Leucine rich peptide (IPR032675)	Leucine-rich repeat neuronal protein 3
Pv_sp_2386.t1	Protein disulfide-isomerase (IPR005792)	Protein disulfide-isomerase A6
Pv_sp_4414.t1	Protein disulfide-isomerase (IPR005792)	Protein disulfide-isomerase
Pv_so_5747.t1	Tachylectin 5A (IPR002181)	Fibrinogen C domain-containing protein 1
Pv_tr_2163.t1	Tachylectin 5A (IPR002181)	Fibrinogen C domain-containing protein 1
Pv_so_2969.t1	Tachylectin 5A (IPR002181)	Fibrinogen C domain-containing protein 1
Pv_sp_4498.t1	Tachylectin 5A (IPR002181)	Fibrinogen C domain-containing protein 1
Pv_tr_15765.t1	Leucine rich peptide (IPR032675)	Leucine-rich repeat transmembrane neuronal protein 1

Table S5. Summary of the toxin precursors with pro-peptide associated sequences, from the merge transcriptome of *Pamphobeteus verdolaga* obtained with the BLAST (Arachnoserver + ToxProt) and HMM (hmmcompete) prediction strategies.

sequence_id	HMM family_name	Blast2GO annotation
Pv_sp_13737.t1	Magi-1 superfamily, ICK 7 family (IPR012627;PF08092;)	NA
Pv_sp_2911.t1	Magi-1 superfamily, HWTX XVIc family (IPR012627;PF08092;)	NA
Pv_sp_13425.t1	Helical arthropod neuropeptide derived (HAND) family	Zinc finger protein 2
Pv_sp_7755.t1	Latarcin superfamily, Lt1 family (IPR018802;PF10279;)	NA
Pv_sp_15140.t1	Huwentoxin-1 family, ProTx 1 subfamily (IPR011696;PF07740;)	NA
Pv_tr_4168.t1	Huwentoxin-1 family, F4a subfamily (IPR011696;PF07740;)	NA
Pv_sp_12914.t1	Protein disulfide-isomerase (IPR005792)	Thioredoxin
Pv_sp_4182.t1	Latarcin superfamily, latarcin lt4 typ2 (IPR018802;PF10279;)	NA
Pv_so_7191.t1	Huwentoxin-1 family, Jztx 15 subfamily (IPR011696;PF07740;)	NA
Pv_sp_14895.t1	Huwentoxin-1 family, HpTx1 subfamily (IPR011696;PF07740;)	NA
Pv_tr_16201.t1	cstx38	Zinc finger and BTB domain-containing protein 49
Pv_sp_6690.t1	Latrotoxin superfamily, Alpha latrocrustotoxin family (IPR002110;IPR020683;PF00023)	Osteoclast-stimulating factor 1
Pv_sp_14524.t1	Cupiennin family, 1 subfamily	NA
Pv_sp_13936.t1	Venom Kunitz-type family, Long Kunitz domain containing protein (IPR002223;IPR020901;PF00014;)	NA
Pv_sp_6028.t1	Huwentoxin-1 family, Jztx 13 subfamily (IPR011696;PF07740;)	NA
Pv_tr_12299.t1	Latrotoxin superfamily, Alpha latrocrustotoxin family (IPR002110;IPR020683;PF00023)	Integrin-linked protein kinase
Pv_sp_14414.t1	CsTx-20	Diphthamide biosynthesis protein 3
Pv_tr_10321.t1	Latrotoxin superfamily, Delta latroinsectotoxin family	Ankyrin repeat domain-containing protein 54
Pv_tr_10510.t1	Venom Serine protease (IPR001314;IPR001254)	Prothrombin
Pv_so_1499.t1	Latrotoxin superfamily, Alpha latrocrustotoxin family (IPR002110;IPR020683;PF00023)	Ankyrin repeat domain-containing protein 65
Pv_tr_7995.t1	Spider agouti family (IPR007733;IPR027300;PF05039;)	NA
Pv_sp_13194.t1	Huwentoxin-1 family, Jztx 36 subfamily (IPR011696;PF07740;)	NA
Pv_so_2810.t1	Huwentoxin-1 family, Jztx 21 subfamily	NA
Pv_sp_9338.t1	Latrotoxin superfamily, Alpha latrocrustotoxin family (IPR002110;IPR020683;PF00023)	Acyl-CoA-binding domain-containing protein 6
Pv_sp_2895.t1	Huwentoxin-1 family, Hntx 13 subfamily (IPR011696;PF07740;)	NA
Pv_sp_14139.t1	Latrotoxin superfamily, Alpha latroinsectotoxin family (IPR002110;IPR020683;PF00023)	Myotrophin
Pv_tr_12363.t1	Huwentoxin-1 family, ProTx III subfamily (IPR011696;PF07740;)	NA
Pv_tr_16488.t1	Latrotoxin superfamily, Alpha latrotoxin family (IPR002110;IPR020683;IPR013829;PF00023)	Ankyrin repeat domain-containing protein 2B
Pv_sp_8040.t1	Spider Agouti	NA
Pv_so_4181.t1	Protein disulfide-isomerase (IPR005792)	Nucleoredoxin
Pv_so_1170.t1	Shiva Omega superfamily, Omega toxin family (IPR009415;PF06357;)	Zinc finger protein 225
Pv_tr_15975.t1	Cysteine rich secretory protein type 0	Golgi-associated plant pathogenesis-related protein 1
Pv_so_3587.t1	Leucine rich peptide (IPR032675)	Leucine-rich repeat protein soc-2 homolog
Pv_so_2925.t1	Latrotoxin superfamily, Delta latroinsectotoxin family	Nuclear factor NF-kappa-B p105 subunit

Pv_so_1535.t1	Latrotoxin superfamily, Alpha latrocrustotoxin family (IPR002110;IPR020683;PF00023)	Ankyrin repeat and SAM domain-containing protein 3
Pv_so_2617.t1	Protein disulfide-isomerase (IPR005792)	Nucleoredoxin
Pv_so_2795.t1	Venom Serine protease (IPR001314;IPR001254)	Transmembrane protease serine 7
Pv_sp_10463.t1	Protein disulfide-isomerase (IPR005792)	Translationally-controlled tumor protein homolog
Pv_tr_6629.t1	MIT-like CsTx-22	NA
Pv_so_4644.t1	Latrotoxin superfamily, Alpha latrotoxin family (IPR002110;IPR020683;IPR013829;PF00023)	Protein fem-1 homolog C
Pv_tr_16263.t1	Spider toxin Tx2 family, OAIP 2 subfamily (IPR004214;PF02950;)	NA
Pv_so_4384.t1	Tachylectin 5A (IPR002181)	Angiopoietin-1
Pv_so_2168.t1	Latrotoxin superfamily, Delta latroinsectotoxin family	Serine/threonine-protein phosphatase 6 regulatory ankyrin repeat subunit B
Pv_tr_10358.t1	Leucine rich peptide (IPR032675)	Leucine-rich repeat transmembrane protein FLRT3
Pv_so_2871.t1	Venom Serine protease (IPR001314;IPR001254)	Vitamin K-dependent protein C
Pv_so_5010.t1	Cysteine rich secretory protein type 1 (IPR014044;IPR002413)	GLIPR1-like protein 1
Pv_sp_2976.t1	Omega-agatoxin superfamily, Type II III omega agatoxin family (IPR005853;IPR013605;PF08396;)	NA
Pv_sp_9706.t1	Protein disulfide-isomerase (IPR005792)	Phosducin-like protein 2
Pv_tr_293.t1	Huwentoxin-1 family, Hntx 8 subfamily (IPR011696;PF07740;)	Arginyl-tRNA--protein transferase 1
Pv_sp_7974.t1	Thyroglobulin-like protein	Thyroglobulin
Pv_tr_12046.t1	Latrotoxin superfamily, Alpha latrocrustotoxin family (IPR002110;IPR020683;PF00023)	Ankyrin-3
Pv_sp_3206.t1	Huwentoxin-1 family, Jztx 9 subfamily (IPR011696;PF07740;)	Zinc finger protein 143
Pv_sp_10034.t1	Cysteine rich secretory protein type 1	Golgi-associated plant pathogenesis-related protein 1
Pv_sp_11473.t1	cstx38	Gastrula zinc finger protein XlCGF57.1
Pv_sp_5365.t1	Huwentoxin-1 family, F4b subfamily (IPR011696;PF07740;)	Ubiquitin domain-containing protein 1
Pv_sp_10087.t1	Protein disulfide-isomerase (IPR005792)	Thioredoxin-related transmembrane protein 2
Pv_sp_6233.t1	Latarcin superfamily, latarcin lt4 typ2 (IPR018802;PF10279;)	Electron transfer flavoprotein subunit alpha
Pv_so_6467.t1	Latarcin superfamily, Lt7 family (IPR018802;PF10279;)	Endothelin-converting enzyme 1
Pv_tr_11557.t1	Latrotoxin superfamily, Alpha latrocrustotoxin family (IPR002110;IPR020683;PF00023)	Serine/threonine-protein phosphatase 6 regulatory ankyrin repeat subunit C
Pv_tr_12300.t1	Latrotoxin superfamily, Alpha latrocrustotoxin family (IPR002110;IPR020683;PF00023)	Ankyrin-3
Pv_sp_6882.t1	Protein disulfide-isomerase (IPR005792)	Protein disulfide-isomerase TMX3
Pv_so_4811.t1	Latrotoxin superfamily, Alpha latrotoxin family (IPR002110;IPR020683;IPR013829;PF00023)	Ankyrin repeat and SOCS box protein 3
Pv_tr_16804.t1	Latrotoxin superfamily, Delta latroinsectotoxin family	Ankyrin repeat and SOCS box protein 13
Pv_tr_2249.t1	cstx38	Zinc finger protein 436
Pv_so_6543.t1	Huwentoxin-1 family, Hntx 9 subfamily (IPR011696;PF07740;)	WD repeat-containing protein 5B
Pv_sp_7373.t1	Latrotoxin superfamily, Alpha latrocrustotoxin family (IPR002110;IPR020683;PF00023)	Ankyrin-3
Pv_tr_17368.t1	Latrotoxin superfamily, Alpha latrocrustotoxin family (IPR002110;IPR020683;PF00023)	Ankyrin-3
Pv_so_6587.t1	Latrotoxin superfamily, Alpha latrocrustotoxin family (IPR002110;IPR020683;PF00023)	Ankyrin repeat and SOCS box protein 11
Pv_so_6201.t1	Leucine rich peptide (IPR032675)	Leucine-rich repeat transmembrane neuronal protein 1
Pv_tr_16681.t1	Venom Serine protease (IPR001314;IPR001254)	Transmembrane protease serine 11B-like protein
Pv_sp_8099.t1	Huwentoxin-1 family, Jztx 21 subfamily	NA
Pv_sp_8012.t1	Huwentoxin-1 family, Hntx 3 subfamily (IPR011696;PF07740;)	FAM8A1

Pv_sp_8234.t1	Tachylectin 5A (IPR002181)	Fibrinogen C domain-containing protein 1
Pv_tr_15867.t1	Latrotoxin superfamily, Alpha latroinsectotoxin family (IPR002110;IPR020683;PF00023)	Ankyrin-3
Pv_tr_13675.t1	Latrotoxin superfamily, Alpha latrocrustotoxin family (IPR002110;IPR020683;PF00023)	Serine/threonine-protein phosphatase 6 regulatory ankyrin repeat subunit B
Pv_sp_8136.t1	Tachylectin 5A (IPR002181)	Fibrinogen C domain-containing protein 1
Pv_so_6474.t1	Latrotoxin superfamily, Alpha latrocrustotoxin family (IPR002110;IPR020683;PF00023)	Ankyrin repeat and SOCS box protein 7
Pv_tr_8828.t1	Latrotoxin superfamily, Alpha latrocrustotoxin family (IPR002110;IPR020683;PF00023)	Ankyrin repeat and SOCS box protein 2
Pv_sp_5082.t1	Protein disulfide-isomerase (IPR005792)	Thioredoxin
Pv_tr_3696.t1	Latrotoxin superfamily, Alpha latrotoxin family (IPR002110;IPR020683;IPR013829;PF00023)	Serine/threonine-protein phosphatase 6 regulatory ankyrin repeat subunit B
Pv_sp_7286.t1	Protein disulfide-isomerase (IPR005792)	DnaJ homolog subfamily C member 10
Pv_tr_9561.t1	Latrotoxin superfamily, Alpha latrotoxin family (IPR002110;IPR020683;IPR013829;PF00023)	Ankyrin-3
Pv_sp_8525.t1	Tachylectin 5A (IPR002181)	Fibrinogen C domain-containing protein 1
Pv_sp_4874.t1	Magi-1 superfamily, ICK 30 40 family (IPR012627;PF08092;)	Protein real-time
Pv_sp_7694.t1	Tachylectin 5A (IPR002181)	Fibrinogen C domain-containing protein 1
Pv_sp_180.t1	Latrotoxin superfamily, Alpha latroinsectotoxin family (IPR002110;IPR020683;PF00023)	Tonsoku-like protein
Pv_sp_4490.t1	Leucine rich peptide (IPR032675)	Leucine-rich repeat transmembrane neuronal protein 3
Pv_sp_7366.t1	Latrotoxin superfamily, Alpha latrocrustotoxin family (IPR002110;IPR020683;PF00023)	Ankyrin repeat and SOCS box protein 5
Pv_tr_9553.t1	Latrotoxin superfamily, Alpha latrocrustotoxin family (IPR002110;IPR020683;PF00023)	Ankyrin-3
Pv_sp_5970.t1	CsTx-29	Ubiquitin-conjugating enzyme E2 Q2
Pv_sp_5164.t1	Leucine rich peptide (IPR032675)	Chondroadherin-like protein
Pv_tr_7820.t1	Latrotoxin superfamily, Alpha latrocrustotoxin family (IPR002110;IPR020683;PF00023)	Ankyrin repeat and MYND domain-containing protein 2
Pv_tr_10074.t1	Latrotoxin superfamily, Alpha latrocrustotoxin family (IPR002110;IPR020683;PF00023)	Ankyrin repeat domain-containing protein 16
Pv_tr_16230.t1	MIT-like AcTx family (IPR020202;PF17556)	Endothelin-converting enzyme 1
Pv_tr_6219.t1	Latrotoxin superfamily, Alpha latrocrustotoxin family (IPR002110;IPR020683;PF00023)	Ankyrin-3
Pv_sp_3877.t1	Latrotoxin superfamily, Alpha latrocrustotoxin family (IPR002110;IPR020683;PF00023)	NF-kappa-B inhibitor cactus
Pv_tr_14031.t1	Cupiennin family, cupiennin typ2	Vacuolar protein sorting-associated protein 8
Pv_tr_2557.t1	Latrotoxin superfamily, Alpha latrocrustotoxin family (IPR002110;IPR020683;PF00023)	Nephrocystin-3
Pv_sp_2305.t1	Latrotoxin superfamily, Delta latroinsectotoxin family	Ankyrin repeat domain-containing protein 13C
Pv_sp_5234.t1	Shiva kappa superfamily (IPR012499;PF07945;)	Phosphatidylinositol 4-kinase type 2-beta
Pv_sp_4907.t1	Latrotoxin superfamily, Alpha latrocrustotoxin family (IPR002110;IPR020683;PF00023)	Ankyrin-3
Pv_sp_3683.t1	Protein disulfide-isomerase (IPR005792)	Protein disulfide-isomerase TMX3
Pv_sp_2220.t1	Latrotoxin superfamily, Alpha latrotoxin family (IPR002110;IPR020683;IPR013829;PF00023)	Ankyrin-3
Pv_tr_9560.t1	Latrotoxin superfamily, Alpha latroinsectotoxin family (IPR002110;IPR020683;PF00023)	Serine/threonine-protein phosphatase 6 regulatory ankyrin repeat subunit C
Pv_so_7596.t1	Latrotoxin superfamily, Alpha latrotoxin family (IPR002110;IPR020683;IPR013829;PF00023)	Integrin-linked protein kinase
Pv_sp_1084.t1	Venom Serine protease (IPR001314;IPR001254)	Prothrombin
Pv_so_7784.t1	Latrotoxin superfamily, Delta latroinsectotoxin family	Serine/threonine-protein phosphatase 6 regulatory ankyrin repeat subunit B
Pv_tr_9552.t1	Latrotoxin superfamily, Alpha latroinsectotoxin family (IPR002110;IPR020683;PF00023)	Serine/threonine-protein phosphatase 6 regulatory ankyrin repeat subunit C
Pv_sp_4749.t1	Protein disulfide-isomerase (IPR005792)	Protein disulfide-isomerase A5
Pv_sp_3775.t1	Leucine rich peptide (IPR032675)	Phenylalanine--tRNA ligase beta subunit

Pv_sp_811.t1	Protein disulfide-isomerase (IPR005792)	Protein disulfide-isomerase
Pv_sp_2149.t1	Latrotoxin superfamily, Delta latroinsectotoxin family	Ankyrin-3
Pv_tr_11868.t1	Latrotoxin superfamily, Alpha latrocrustotoxin family (IPR002110;IPR020683;PF00023)	Serine/threonine-protein phosphatase 6 regulatory ankyrin repeat subunit B
Pv_sp_4722.t1	Venom Serine protease (IPR001314;IPR001254)	Inactive serine protease PAMR1
Pv_tr_15236.t1	Leucine rich peptide (IPR032675)	Leucine-rich repeat-containing protein 40
Pv_tr_16028.t1	Latrotoxin superfamily, Alpha latrocrustotoxin family (IPR002110;IPR020683;PF00023)	Protein phosphatase 1 regulatory subunit 12B
Pv_so_7815.t1	Latrotoxin superfamily, Alpha latrocrustotoxin family (IPR002110;IPR020683;PF00023)	Ankyrin repeat domain-containing protein 13C
Pv_so_7889.t1	Insecticidal toxin ABC family, Calisoga subfamily (IPR012626;PF08091;)	BRCA1-associated ATM activator 1
Pv_sp_2806.t1	Latrotoxin superfamily, Alpha latrocrustotoxin family (IPR002110;IPR020683;PF00023)	Ankyrin repeat, SAM and basic leucine zipper domain-containing protein 1
Pv_sp_2345.t1	Latrotoxin superfamily, Delta latroinsectotoxin family	Chaperone protein ClpB
Pv_tr_4893.t1	Leucine rich peptide (IPR032675)	Leucine-rich repeat protein soc-2 homolog
Pv_tr_4993.t1	Leucine rich peptide (IPR032675)	Insulin-like growth factor-binding protein complex acid labile subunit
Pv_tr_9244.t1	Latrotoxin superfamily, Alpha latrotoxin family (IPR002110;IPR020683;IPR013829;PF00023)	SH3 and multiple ankyrin repeat domains protein 3
Pv_tr_16673.t1	Leucine rich peptide (IPR032675)	Platelet glycoprotein V
Pv_sp_1011.t1	Latrotoxin superfamily, Alpha latroinsectotoxin family (IPR002110;IPR020683;PF00023)	Ankyrin repeat and IBR domain-containing protein 1
Pv_sp_1671.t1	Latrotoxin superfamily, Alpha latrocrustotoxin family (IPR002110;IPR020683;PF00023)	Protein fem-1 homolog C
Pv_so_8281.t1	Tachylectin 5A (IPR002181)	Endothelin-converting enzyme 1
Pv_sp_2016.t1	Latarcin superfamily, Lt7 family (IPR018802;PF10279;)	Endothelin-converting enzyme 1
Pv_tr_4977.t1	Peptidylglycine alpha-amidating monooxygenase (IPR000720)	Endothelin-converting enzyme 1
Pv_tr_9073.t1	Venom Kunitz-type family, Long Kunitz domain containing protein (IPR002223;IPR020901;PF00014;)	Papilin
Pv_tr_15777.t1	Latarcin superfamily, Lt7 family (IPR018802;PF10279;)	Endothelin-converting enzyme 1
Pv_sp_1844.t1	Latrotoxin superfamily, Alpha latroinsectotoxin family (IPR002110;IPR020683;PF00023)	Neprilysin
Pv_tr_5050.t1	Latrotoxin superfamily, Alpha latroinsectotoxin family (IPR002110;IPR020683;PF00023)	Endothelin-converting enzyme 1
Pv_tr_1311.t1	Latrotoxin superfamily, Alpha latrocrustotoxin family (IPR002110;IPR020683;PF00023)	Protein phosphatase 1 regulatory subunit 12A
Pv_sp_965.t1	Spider Agouti	Solute carrier organic anion transporter family member 1A5
Pv_sp_1085.t1	Latrotoxin superfamily, Alpha latrocrustotoxin family (IPR002110;IPR020683;PF00023)	Protein phosphatase 1 regulatory subunit 12B
Pv_tr_15800.t1	Latrotoxin superfamily, Alpha latrotoxin family (IPR002110;IPR020683;IPR013829;PF00023)	Histone-lysine N-methyltransferase SUV39H2
Pv_sp_807.t1	Latrotoxin superfamily, Delta latroinsectotoxin family	Apoptosis-stimulating of p53 protein 1
Pv_tr_305.t1	Latrotoxin superfamily, Alpha latrocrustotoxin family (IPR002110;IPR020683;PF00023)	Death-associated protein kinase 1
Pv_sp_1095.t1	Latrotoxin superfamily, Alpha latrocrustotoxin family (IPR002110;IPR020683;PF00023)	Oxysterol-binding protein-related protein 1
Pv_tr_17643.t1	Latrotoxin superfamily, Alpha latrocrustotoxin family (IPR002110;IPR020683;PF00023)	Serine/threonine-protein phosphatase 6 regulatory ankyrin repeat subunit C
Pv_tr_16469.t1	Latrotoxin superfamily, Alpha latrotoxin family (IPR002110;IPR020683;IPR013829;PF00023)	Histone-lysine N-methyltransferase SUV39H2
Pv_sp_885.t1	Latrotoxin superfamily, Alpha latroinsectotoxin family (IPR002110;IPR020683;PF00023)	Ankyrin-3
Pv_so_8457.t1	Latrotoxin superfamily, Alpha latroinsectotoxin family (IPR002110;IPR020683;PF00023)	Inhibitor of Bruton tyrosine kinase
Pv_sp_431.t1	Latrotoxin superfamily, Alpha latrotoxin family (IPR002110;IPR020683;IPR013829;PF00023)	Ankyrin-3
Pv_tr_15558.t1	Latrotoxin superfamily, Alpha latroinsectotoxin family (IPR002110;IPR020683;PF00023)	Ankyrin-3
Pv_sp_359.t1	Latrotoxin superfamily, Alpha latrocrustotoxin family (IPR002110;IPR020683;PF00023)	Ankyrin-3
Pv_sp_364.t1	Latrotoxin superfamily, Alpha latrocrustotoxin family (IPR002110;IPR020683;PF00023)	Protein TANC2

Pv_tr_5723.t1	Latrotoxin superfamily, Alpha latroinsectotoxin family (IPR002110;IPR020683;PF00023)	Kinesin light chain 4
Pv_tr_3655.t1	Latrotoxin superfamily, Alpha latroinsectotoxin family (IPR002110;IPR020683;PF00023)	Poly [ADP-ribose] polymerase 1
Pv_sp_63.t1	Latrotoxin superfamily, Alpha latroinsectotoxin family (IPR002110;IPR020683;PF00023)	Ankyrin-3
Pv_tr_10178.t1	Latrotoxin superfamily, Alpha latrotoxin family (IPR002110;IPR020683;IPR013829;PF00023)	Ankyrin-3
Pv_sp_4.t1	Latrotoxin superfamily, Delta latroinsectotoxin family	Ankyrin-3

Table S6. Summary of the toxin precursors with neither signal or pro-peptide associated sequences, from the merge transcriptome of *Pamphobeteus verdolaga* obtained with the BLAST (Arachnoserver + ToxProt) and HMM (hmmcompete) prediction strategies.

sequence_id	HMM family_name	Blast2GO annotation
Pv_sp_4102.t1	Huwentoxin-1 family, AU5B subfamily (IPR011696;PF07740;)	NA
Pv_tr_13769.t1	Magi-1 superfamily, ICK 30 40 family (IPR012627;PF08092;)	NA
Pv_tr_726.t1	Latrotoxin superfamily, Alpha latroinsectotoxin family (IPR002110;IPR020683;PF00023)	Myotrophin
Pv_so_1314.t1	Latrotoxin superfamily, Alpha latrotoxin family (IPR002110;IPR020683;IPR013829;PF00023)	Ankyrin repeat domain-containing protein 29
Pv_sp_15275.t1	Huwentoxin-1 family, Jztx F5 subfamily (IPR011696;PF07740;)	NA
Pv_sp_15666.t1	Huwentoxin-1 family, ProTx 1 subfamily (IPR011696;PF07740;)	Neprilysin
Pv_tr_15994.t1	CsTx-35	Ferredoxin-dependent glutamate synthase
Pv_tr_6775.t1	Protein disulfide-isomerase (IPR005792)	Protein disulfide-isomerase A3
Pv_tr_9551.t1	Latrotoxin superfamily, Alpha latrocrustotoxin family (IPR002110;IPR020683;PF00023)	Serine/threonine-protein phosphatase 6 regulatory ankyrin repeat subunit B
Pv_sp_12669.t1	Huwentoxin-1 family, ProTx 1 subfamily (IPR011696;PF07740;)	NA
Pv_so_7116.t1	Spider toxin Tx3-6 family	NA
Pv_so_743.t1	Huwentoxin-1 family, GrTx subfamily (IPR011696;PF07740;)	NA
Pv_sp_13021.t1	Protein disulfide-isomerase (IPR005792)	Thioredoxin
Pv_sp_13996.t1	Huwentoxin-1 family (IPR011696;PF07740;)	U11-theraphotoxin-Cg1b
Pv_so_1664.t1	Thyroglobulin-like protein	Insulin-like growth factor-binding protein 5
Pv_so_2505.t1	Latarcin superfamily, Lt7 family (IPR018802;PF10279;)	NA
Pv_tr_2354.t1	Protein disulfide-isomerase (IPR005792)	Protein disulfide-isomerase
Pv_so_3857.t1	Latarcin superfamily, Lt6 family (IPR018802;PF10279;)	NA
Pv_so_3471.t1	Huwentoxin-1 family, ProTx 1 subfamily (IPR011696;PF07740;)	Endothelin-converting enzyme 1
Pv_tr_638.t1	Huwentoxin-1 family, ScTx1 subfamily (IPR011696;PF07740;)	Protein O-mannose kinase
Pv_tr_11130.t1	Angiotensin-converting Enzyme (IPR033591)	Angiotensin-converting enzyme
Pv_tr_4717.t1	Venom Serine protease (IPR001314;IPR001254)	Plasma kallikrein
Pv_sp_12615.t1	Magi-5 family, Omega atracotoxin type 2 family (IPR012628;IPR013139;PF08093;)	NA
Pv_so_4240.t1	Latrotoxin superfamily, Alpha latroinsectotoxin family (IPR002110;IPR020683;PF00023)	Ankyrin repeat domain-containing protein 39
Pv_so_4504.t1	Latrotoxin superfamily, Alpha latrocrustotoxin family (IPR002110;IPR020683;PF00023)	Ankyrin repeat domain-containing protein 39
Pv_sp_5251.t1	Signal Peptidase (IPR001733)	Signal peptidase complex catalytic subunit SEC11
Pv_tr_10035.t1	Tachylectin 5A (IPR002181)	Fibrinogen C domain-containing protein 1
Pv_tr_3301.t1	Delta-atracotoxin family (IPR008017;PF05353;)	Transposable element Tcb1 transposase

Pv_sp_10768.t1	Insecticidal toxin ABC family, Calisoga subfamily (IPR012626;PF08091;)	NA
Pv_tr_5818.t1	Latrotoxin superfamily, Alpha latroinsectotoxin family (IPR002110;IPR020683;PF00023)	Ankyrin-3
Pv_sp_9101.t1	Latrotoxin superfamily, Alpha latrotoxin family (IPR002110;IPR020683;IPR013829;PF00023)	Integrin-linked protein kinase
Pv_sp_8418.t1	Leucine rich peptide (IPR032675)	U2 small nuclear ribonucleoprotein A
Pv_sp_8686.t1	Latrotoxin superfamily, Alpha latroinsectotoxin family (IPR002110;IPR020683;PF00023)	Serine/threonine-protein phosphatase 6 regulatory ankyrin repeat subunit C
Pv_tr_16664.t1	Huwentoxin-2 family, TXP1 subfamily (IPR012625;PF08089;)	Protein suppressor of hairy wing
Pv_sp_6602.t1	Angiotensin-converting Enzyme (IPR033591)	Acetylcholinesterase
Pv_tr_17006.t1	Cstx-37	RNA-directed DNA polymerase from mobile element jockey
Pv_tr_5896.t1	Latarcin superfamily, Lt7 family (IPR018802;PF10279;)	Endothelin-converting enzyme 1
Pv_tr_6699.t1	Latrotoxin superfamily, Alpha latrotoxin family (IPR002110;IPR020683;IPR013829;PF00023)	Ankyrin repeat and SOCS box protein 2
Pv_sp_4842.t1	Protein disulfide-isomerase (IPR005792)	Glutaredoxin-3
Pv_so_7311.t1	Latrotoxin superfamily, Alpha latrocrustotoxin family (IPR002110;IPR020683;PF00023)	Ankyrin-3
Pv_tr_9705.t1	Venom Serine protease (IPR001314;IPR001254)	Prostasin
Pv_sp_2780.t1	Leucine rich peptide (IPR032675)	Insulin-like growth factor-binding protein complex acid labile subunit
Pv_so_7402.t1	Double-knot toxin subfamily	Dynein assembly factor with WDR repeat domains 1
Pv_sp_3698.t1	Shiva kappa superfamily (IPR012499;PF07945;)	Phosphatidylinositol 4-kinase type 2-beta
Pv_sp_3431.t1	Latrotoxin superfamily, Alpha latrocrustotoxin family (IPR002110;IPR020683;PF00023)	Speckle-type POZ protein
Pv_tr_2037.t1	Latarcin superfamily, Lt7 family (IPR018802;PF10279;)	Myocardin
Pv_tr_10546.t1	Protein disulfide-isomerase (IPR005792)	Endothelin-converting enzyme 1
Pv_tr_6330.t1	Leucine rich peptide (IPR032675)	Thyroid peroxidase
